# Convergence of Thermistor Materials and Focal Plane Arrays in Uncooled Microbolometers: Trends and Perspectives

**DOI:** 10.3390/nano15171316

**Published:** 2025-08-27

**Authors:** Bo Wang, Xuewei Zhao, Tianyu Dong, Ben Li, Fan Zhang, Jiale Su, Yuhui Ren, Xiangliang Duan, Hongxiao Lin, Yuanhao Miao, Henry H. Radamson

**Affiliations:** Research and Development Center of Optoelectronic Hybrid IC, Guangdong Greater Bay Area Institute of Integrated Circuit and System, Guangzhou 510535, China; wangbo@giics.com.cn (B.W.); linhongxiao@giics.com.cn (H.L.)

**Keywords:** uncooled microbolometers, thermistor materials, FPAs, ultra-high resolution, infrared detection

## Abstract

Uncooled microbolometers play a pivotal role in infrared detection owing to their compactness, low power consumption, and cost-effectiveness. This review comprehensively summarizes recent progress in thermistor materials and focal plane arrays (FPAs), highlighting improvements in sensitivity and integration. Vanadium oxide (VO_x_) remains predominant, with Al-doped films via atomic layer deposition (ALD) achieving a temperature coefficient of resistance (TCR) of −4.2%/K and significant *1/f* noise reduction when combined with single-walled carbon nanotubes (SWCNTs). Silicon-based materials, such as phosphorus-doped hydrogenated amorphous silicon (α-Si:H), exhibit a TCR exceeding −5%/K, while titanium oxide (TiO_x_) attains TCR values up to −7.2%/K through ALD and annealing. Emerging materials including GeSn alloys and semiconducting SWCNT networks show promise, with SWCNTs achieving a TCR of −6.5%/K and noise equivalent power (NEP) as low as 1.2 mW/√Hz. Advances in FPA technology feature pixel pitches reduced to 6 μm enabled by vertical nanotube thermal isolation, alongside the 3D heterogeneous integration of single-crystalline Si-based materials with readout circuits, yielding improved fill factors and responsivity. State-of-the-art VO_x_-based FPAs demonstrate noise equivalent temperature differences (NETD) below 30 mK and specific detectivity (D*) near 2 × 10^10^ cm⋅Hz ^1/2^/W. Future advancements will leverage materials-driven innovation (e.g., GeSn/SWCNT composites) and process optimization (e.g., plasma-enhanced ALD) to enable ultra-high-resolution imaging in both civil and military applications. This review underscores the central role of material innovation and system optimization in propelling microbolometer technology toward ultra-high resolution, high sensitivity, high reliability, and broad applicability.

## 1. Introduction

**Figure 1:**
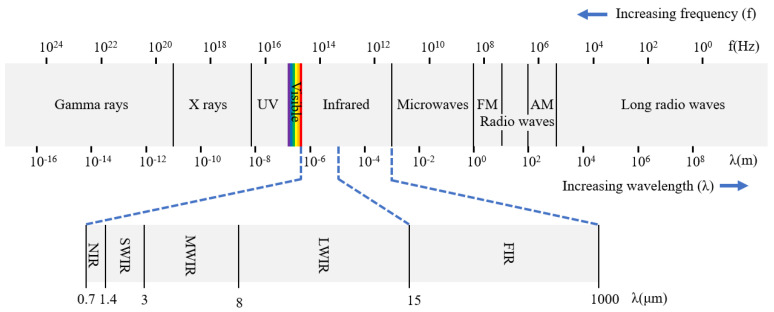
Electromagnetic spectrum with sub-regions.

The infrared (IR) spectrum lies between the visible and microwave regions, with a wavelength range extending from approximately 0.78 to 1000 μm. This broad spectrum is subdivided into several distinct sub-bands, including near-infrared (NIR) in the 0.78 to 1.4 μm range [1,2,3,4,5,6], short-wave infrared (SWIR) from 1.4 to 3 μm [7,8,9,10,11,12,13,14,15,16,17], mid-wave infrared (MWIR) from 3 to 8 μm [18,19,20,21,22,23], long-wave infrared (LWIR) from 8 to 15 μm [24,25,26,27,28,29,30], and far-infrared (FIR) from 15 to 1000 μm [31,32,33,34,35,36] (Figure 1). Infrared detectors are typically categorized based on their detection mechanisms into two primary types: photon infrared detectors [37,38,39,40,41,42] and thermal infrared detectors [43,44,45,46,47,48,49,50,51,52].

Compared to cooled infrared detectors, uncooled infrared detectors offer several distinct advantages, mainly due to their ability to operate at ambient temperatures without requiring cryogenic cooling. This feature leads to reduced system size and weight, lower power consumption, improved reliability and operational lifespan, and decreased manufacturing and maintenance costs, as well as faster startup times [53,54]. Although uncooled infrared detectors generally exhibit lower sensitivity than their cooled counterparts, their performance is often sufficient to meet the technical requirements of a wide range of military and civilian applications. Currently, the production volume of uncooled detectors far exceeds that of cooled detectors, driven primarily by rising demand in the civilian sector. Continuous technological advancements have also enabled uncooled detectors to fulfill specific military application needs [55,56,57,58]. Among the various types of uncooled thermal detectors, microbolometers have emerged as the dominant technology, offering a compelling combination of sensitivity, compactness, and cost-effectiveness. Microbolometers operate based on the temperature-dependent resistance change of thermistor materials: incident infrared radiation absorbed by the detector raises the temperature of the thermistor element, causing a measurable resistance change, which is typically a decrease with increasing temperature, according to the material’s intrinsic properties [59].

Based on the choice of thermistor materials, microbolometers can be broadly categorized into VO_x_ microbolometers and α-Si microbolometers. Among them, VO_x_ microbolometers are the most widely adopted in uncooled infrared detection systems, primarily due to their relatively high temperature coefficient of resistance (TCR), typically in the range of −2% to −3%/K, which enables enhanced sensitivity. However, VOx materials are incompatible with standard CMOS fabrication processes and must be produced on dedicated lines to avoid contamination, thereby increasing the production complexity and cost. To address these challenges, α-Si-based based microbolometers have been proposed as a promising alternative, owing to the following advantages: (i) full compatibility with standard CMOS processes, which not only improves the fabrication yield and operational reliability but also facilitates low-cost large-scale manufacturing; (ii) comparable TCR values to those of VO_x_, along with the ability to be deposited using plasma-enhanced chemical vapor deposition (PECVD). This technique supports large-area, low-temperature (below 350 °C), and uniform film deposition, making it suitable for high-throughput production. Nonetheless, α-Si microbolometers also face several drawbacks, such as elevated *1/f* noise levels, a more intricate film formation process, and susceptibility to photo-induced degradation effects, commonly referred to as the Staebler–Wronski (SW) effect.

With the advancement of infrared technology, the market has imposed increasingly stringent requirements on the sensitivity of uncooled microbolometers and the resolution of infrared imaging. Various alternative thermistor materials are under investigation, including polymorphous silicon–germanium alloy (pm-Si_x_Ge_1−x_:H) [60,61,62,63], α-Si_x_Ge_y_O_1−x−y_ films [64,65,66,67,68], crystalline SiGe/Si quantum wells (QWs) [69,70,71,72,73,74], crystalline GeSi/Ge QWs [75], Ge quantum dots [76], TiO_x_ [77,78,79], amorphous germanium–tin (Ge_1−x_Sn_x_), amorphous germanium [80], amorphous nickel oxide (Ni_1−x_O) [81], and carbon nanotubes [82,83,84,85]. Along with advances in thermistor materials, the process flows and pixel microstructures of microbolometers have also undergone significant improvements. The exploration of diverse thermistor materials, process technologies, and pixel architectures is crucial to the ongoing evolution of microbolometer technology, holding great promise for meeting increasingly demanding market requirements and pushing infrared detection and imaging to new performance levels. Driven by the rapid development of uncooled infrared imaging technology over the past few decades, the applications of microbolometers have expanded beyond the military domain into a wide range of civilian and commercial sectors. These include night vision [86,87,88], surveillance [89,90,91,92], security systems [93], threat detection [94,95,96], agriculture [97,98,99,100,101], firefighting [102,103,104,105], search and rescue [106,107], leak detection [108,109,110,111,112], gas analysis [113], process monitoring [114,115,116], industrial inspection [117,118], medical diagnostics [119,120,121,122,123], driving assistance [124,125,126,127], and consumer electronics [128,129,130].

In the field of microbolometer research, previous reviews have primarily addressed the conventional aspects of microbolometer technology [131,132,133,134,135,136,137]. However, recent developments including novel thermistor materials such as Al-doped VO_x_ and semiconducting single-walled carbon nanotube (SWCNT) networks, advanced fabrication techniques like three-dimensional heterogeneous integration and plasma-enhanced ALD, as well as innovative pixel architectures such as vertical nanotube thermal isolation have significantly expanded device capabilities and underscore the need for a comprehensive update. This review offers a unique synthesis by integrating material–property optimization, process-technology innovation, and system-level performance evaluation. It critically examines thermistor materials from the perspective of defect engineering to overall device performance, explores fabrication strategies that enable CMOS compatibility with emerging materials such as single-crystalline SiGe, and highlights recent advances in pixel design and system integration. Unlike previous works, the focus here is on the synergistic effects of co-optimizing materials and processes, emphasizing their collective impact on key performance parameters including TCR, responsivity, and noise characteristics, supported by recent experimental findings. The review begins by revisiting the fundamental operating principles with an updated framework for performance assessment, followed by detailed discussions on cutting-edge thermistor materials and focal plane array architectures. The concluding section synthesizes these insights to outline a forward-looking roadmap for advancing microbolometer technology toward higher resolution imaging and emerging applications in fields such as drone-based thermal sensing and wearable diagnostics.

## 2. Fundamentals of Thermal Detectors

### 2.1. Basic Operation Principles

The fundamental purpose of all infrared detectors is to convert the invisible infrared radiation into measurable physical quantities. The bolometer is a resistive element, which is composed of a thermistor material with a large temperature coefficient and a small thermal capacity. It absorbs infrared radiation to heat the thermistor material, thereby causing a change in the pixel resistance value. The detector sensitive element is connected to a constant temperature substrate heat sink through a support structure. Similar to all thermal infrared detectors, analysis of microbolometers begins with the heat balance equation. Although the operation of microbolometers requires bias, the heat balance equation that ignores the Joule power still has important reference significance. The simplified heat balance equation describes the temperature change of microbolometer by using the following expression [138]:

(1) $$C\frac{d(\Delta T)}{dt}+G(\Delta T)=\eta \beta A_{D}P=\eta \beta A_{D}P_{o}\exp(j\omega t),$$

where “*C*” represents the thermal capacity of sensitive element, “Δ*T*” denotes the temperature change of the sensitive element brought about by incident infrared radiation power; “*G*” stands for the thermal coupling of the sensitive element to its surroundings; “*η*” is the optical absorption coefficient of the radiation-sensing material; “*β*” is the fill factor, which determines the portion of the pixel area utilized for absorbing the incident infrared radiation, which is equivalent to the ratio of the pixel effective area to the total pixel area; “*A*_D_” indicates the total area of the pixel; “*P*_0_” is the power amplitude of the temporally modulated infrared radiation that impinges on the pixel; “*j*” is defined as √−1; “*ω*” represents the angular frequency of radiation modulation; “*t*” represents the time. The expression pertaining to the temperature rise can be derived by solving the heat balance equation [138,139,140]:

(2) $$\Delta T=\frac{\eta \beta A_{D}P_{0}\exp(j\omega t)}{G+j\omega C}=\frac{\eta \beta A_{D}P_{0}}{G{\left(1+\omega^{2}\tau^{2}\right)}^{1/2}}.$$

Here, *τ* represents the thermal response time, which is defined by the following equation [138]:

(3) $$\tau =\frac{C}{G}.$$

The thermal response time, alternatively referred to as the thermal time constant, represents the decay time or rise time necessary for the detector signal to attain the value of 1/e or (1–1/e) of the incident radiated rectangular wave pulse, respectively [141]. This parameter serves as a crucial metric for evaluating the speed and sensitivity of the bolometer. Specifically, it gauges how swiftly the bolometer reacts to the alterations in its temperature during the heating-up and cooling-down (thermal relaxation) cycles. Ideally, one aims to align the detector time constant with system frame rate. One common design practice is to engineer the pixel response time to be shorter than one-half of the reciprocal of the system frame rate [142]. The typical response time of a bolometer is approximately 10 ms, which is notably longer than that of infrared photodetector, with the latter typically falling within the microsecond range [131].

As can be deduced from Equation (2), the temperature of the sensitive element undergoes oscillations, increasing when the input infrared radiation power rises and decreasing when it falls. The transition between low-frequency and high-frequency regions is characterized by the thermal response time *τ* [143]. In the low-frequency regime, where *ωτ* << 1, the temperature change remains independent of the frequency, and it can be expressed by the following equation:

(4) $$\Delta T=\frac{\eta \beta A_{D}P_{0}}{G}.$$

In the high-frequency range, where *ωτ* >> 1, the temperature change exhibits an inverse proportionality to the frequency, and it can be represented by the following equation:

(5) $$\Delta T=\frac{\eta \beta A_{D}P_{0}}{\omega C}.$$

In the context of microbolometers, a temperature elevation of 1 K in the object typically leads to a corresponding temperature increase on the order of 4 mK within the bolometer diaphragm [144].

### 2.2. Figures of Merit

To accurately evaluate a bolometer, it is essential to quantitatively describe its performance. The performance of a bolometer is typically benchmarked using several figures of merit, namely the TCR, responsivity (*ℜ*), noise equivalent power (NEP), noise equivalent temperature difference (NETD), and specific detectivity (D*). In this section, the diverse figures of merit associated with bolometers will be presented.

#### 2.2.1. Temperature Coefficient of Resistance (TCR)

The relative variation in the resistance value of the thermoresistive material within a bolometer with respect to temperature is defined as the TCR, which is represented by the symbol α. It can be expressed as follows [145]:

(6) $$\alpha =\frac{1}{R_{b}}\frac{dR_{b}}{dT},$$

where *R*_b_ denotes the resistance of the bolometer, and *T* represents the temperature of the bolometer.

For metal materials, the conduction band and valence band overlap one another. Consequently, the free carrier density of metal materials scarcely changes with the temperature. However, as the temperature rises, the thermal motion of the free carriers intensifies. This leads to an increase in the average velocity of electrons and a decrease in the mean time between collisions. The electrical resistivity of metals increases as the mean time between collisions decreases; in other words, the electrical resistance of metals rises with an increase in temperature. Hence, the TCR values of metals are positive. In contrast to metal materials, semiconductor materials display distinct resistance–temperature characteristics. Some carriers near the top of the semiconductor valence band are excited to the conduction band due to the increased thermal energy. The resulting increase in free carrier density causes a decrease in the resistance of semiconductors. Consequently, semiconductor materials exhibit negative TCR values. A typical resistance–temperature behavior for a semiconductor is presented in [145]:

(7) $$R(T)=R_{0}\exp(\frac{\Delta Ea}{k_{B}T}).$$

By combining Equations (6) and (7), we obtain

(8) $$\alpha =-\frac{\Delta E_{a}}{k_{B}T^{2}}.$$

Here, Δ*E*_a_ represents the activation energy, *k*_B_ denotes the Boltzmann’s constant, and T stands for the temperature of the bolometer. For semiconductor materials, the value of α increases with the increment in Δ*E*_a_. On the other hand, the concentration of free carriers decreases as Δ*E*_a_ increases. Generally speaking, semiconductor materials with higher resistance tend to have a higher value of α. Regrettably, semiconductor materials with high resistance frequently exhibit high *1/f* noise.

#### 2.2.2. Responsivity (ℜ)

The responsivity (ℜ) serves as a metric to gauge the dependence of the signal output of a bolometer on the input radiant power. It is defined as the ratio of the pixel output signal to the input radiant power incident on the pixel, thereby reflecting the efficiency of the device in converting the incident infrared radiation into an electrical signal. The pixel output signal can manifest either as voltage or current. The voltage responsivity (*ℜ*_V_) and the current responsivity (*ℜ*_I_) are defined as follows [138]:

(9) $${ℜ}_{V}=\frac{V_{S}}{P_{0}A_{D}},$$

(10) $${ℜ}_{I}=\frac{I_{S}}{P_{0}A_{D}}.$$

Here, *V*_S_ represents the output signal voltage, *I*_S_ denotes the output signal current, *P*_0_ stands for the radiant power incident upon the pixel, and A_D_ indicates the total area of the pixel. The voltage responsivity (*ℜ*_V_) is expressed in the unit of Volts/Watts, whereas the current responsivity (*ℜ*_I_) is expressed in the unit of Amps/Watts. Assuming that the temperature change of the bolometer resulting from the absorption of infrared radiation is sufficiently small such that the change in resistance exhibits a linear relationship with the change in temperature, then the resistance can be expressed in terms of the TCR (α), namely,

(11) $$\Delta R_{b}=\alpha R_{b}\Delta T.$$

Consequently, expressions for *ℜ*_V_ and *ℜ*_I_ are as follows [135,138,146,147]:

(12) $${ℜ}_{V}=\frac{\eta \beta I_{b}\alpha R_{b}}{G{\left(1+\omega^{2}\tau^{2}\right)}^{1/2}}$$

(13) $${ℜ}_{I}=\frac{\eta \beta V_{b}\alpha }{GR_{b}{\left(1+\omega^{2}\tau^{2}\right)}^{1/2}}.$$

Herein, η represents the infrared radiation absorption coefficient, β denotes the fill factor, *I*_b_ stands for the current bias applied to the bolometer, *V*_b_ indicates the voltage bias applied to the bolometer, *R*_b_ represents the electrical resistance of the bolometer, *G* is the total thermal conductance that couples the bolometer to its surroundings, *ω* is the angular frequency of modulation of the radiation, and *τ* is the thermal response time of the bolometer. The responsivity remains independent of the frequency at low modulation frequencies and decreases with increasing frequency at higher frequencies. Moreover, it exhibits a positive correlation with the TCR (α) and a negative correlation with the thermal conductivity *G*. The crucial aspect in enhancing the responsivity lies in having a high value of the temperature coefficient α along with excellent thermal isolation (low thermal conductance *G*) [148]. Additionally, a low thermal capacity *C* is also requisite, since merely reducing the thermal conductance can result in a significant increase in the thermal response time.

#### 2.2.3. Noise Equivalent Power

However, a high responsivity does not necessarily imply that the bolometer can detect weak optical signal variations, since random noise fluctuations may render the signal indistinguishable. It is essential to employ a more suitable parameter to gauge the ability of the bolometer to extract signals from ambient noise. The NEP is defined as the incident radiant power required to generate a signal equivalent to the root mean square (RMS) of the noise. This represents the minimum level of absorbed optical radiation that the bolometer can detect. The NEP is related to the noise voltage (*V*_N_) and *ℜ* and is usually expressed as [138]

(14) $$NEP=\frac{V_{N}}{{ℜ}_{V}},$$

where *V*_N_ represents the RMS total noise voltage of the bolometer within the system bandwidth. The primary noise sources of the bolometer consist of Johnson noise, *1/f* noise, temperature fluctuation noise, and background fluctuation noise. The total noise voltage is acquired by orthogonally combining the noise voltages of these four independent types of noise contributions and can be expressed as follows [138]:

(15) $$\bar{V_{N}^{2}}=\bar{V_{j}^{2}}+\bar{V_{1/f}^{2}}+\bar{V_{TF}^{2}}+\bar{V_{BF}^{2}}.$$

Johnson noise exists in all resistors and is induced by the random motion of free carriers within the resistive material. It is also referred to as Nyquist thermal noise or simply thermal noise. The expression for the Johnson noise voltage is presented as follows [138]:

(16) $$V_{j}=\sqrt{4k_{B}TR_{b}\Delta f},$$

where *k*_B_ represents the Boltzmann’s constant, *T* stands for the temperature of the bolometer, *R_b_* denotes the resistance of the bolometer, and Δ*f* indicates the electrical bandwidth of the signal processing electronics. It can be observed that the Johnson noise is independent of the bias voltage and operating frequency of the bolometer. It is a type of white noise, and its magnitude increases as the temperature of the bolometer goes up.

Currently, the *1/f* noise of thermal material itself constitutes the largest noise source in uncooled microbolometer systems. Although the precise origin of *1/f* noise remains unknown, it is potentially associated with factors such as the number of carriers, spontaneous temperature fluctuations, and presence of traps or defects in the material [149,150,151]. Multiple factors exert an influence on *1/f* noise, including the thermistor material [152,153], deposition technique [154], surface treatment method [155], and the electrode contact [156,157,158,159], among others. The *1/f* noise voltage can be characterized by the Hooge’s formulation [140,160,161]:

(17) $$V_{1/f}=\sqrt{\frac{K_{1/f}V_{b}^{2}}{f}}\cdot \sqrt{\Delta f}.$$

Herein, *V*_b_ represents the voltage bias applied to the bolometer, f denotes the electrical frequency, Δ*f* indicates the electrical bandwidth of the signal processing electronics, *f*_1_ stands for the cut-on frequency of the amplifier, and *K*_1/f_ is the *1/f* noise parameter, which has the following expression:

(18) $$K_{1/f}=\frac{\alpha_{H}}{N}.$$

Here, α_H_ represents the Hooge parameter, and *N* denotes the total number of free carriers in the thermistor film which is volume dependent. In any thermodynamic system, the temperature undergoes random fluctuations. The expression for the temperature fluctuation is presented as follows:

(19) $$\Delta T_{F}=\frac{4kT^{2}\Delta f}{G{\left(1+\omega^{2}\tau^{2}\right)}^{1/2}}.$$

The temperature fluctuation noise is induced by the random heat exchange occurring between the bolometer and its surrounding environment. The voltage of the temperature fluctuation noise can be expressed by the following equation [162]:

(20) $$V_{TF}=\frac{2I_{b}R_{b}\alpha T\sqrt{\Delta fk_{B}}}{\sqrt{G\left(1+\omega^{2}\tau^{2}\right)}}.$$

Herein, *I_b_* represents the current bias applied to the bolometer, *R_b_* denotes the resistance of the bolometer, *η* stands for the optical absorption coefficient, α is the TCR, *T* is the temperature of the bolometer, Δ*f* indicates the electrical bandwidth of the signal processing electronics, *k*_B_ represents Boltzmann’s constant, *G* is the total thermal conductance that couples the bolometer to its surroundings, ω is the angular frequency of modulation of the radiation, and *τ* is the thermal response time of the bolometer. The background fluctuation noise is induced by the random fluctuations in the radiation exchange taking place between the bolometer and its surrounding environment. The voltage of the background fluctuation noise can be expressed by the following equation [162]:

(21) $$V_{BG}=\frac{I_{b}R_{b}\alpha \sqrt{8A_{D}\eta \sigma k_{B}\left(T^{5}+T_{B}^{5}\right)\Delta f}}{G\sqrt{\left(1+\omega^{2}\tau^{2}\right)}}.$$

Here, *A_D_* represents the total area of the pixel, *η* denotes the optical absorption coefficient, σ is the Stefan–Boltzmann constant, *T* stands for the temperature of the bolometer, and *T_B_* indicates the background temperature. For current noise and current signals, NEP can accordingly be expressed as the following equation:

(22) $$NEP=\frac{I_{N}}{{ℜ}_{I}}.$$

Here, *I_N_* represents the RMS total noise current of the bolometer within the system bandwidth. *NEP* is typically defined at the system bandwidth of 1 Hz and is expressed in units of Watts per square root of Hertz (Watts/Hz^1^/^2^). The *NEP* serves as a metric where a lower value is more favorable, and it can be minimized either by reducing the noise voltage (or noise current) or by enhancing the responsivity.

#### 2.2.4. Noise Equivalent Temperature Difference

The NETD represents the most crucial figure of merit within infrared sensing systems, which is defined as the temperature change at the target that gives rise to a signal-to-noise ratio of one [163]. Essentially, it is the minimum temperature difference that can be detected by a bolometer and is commonly expressed in the unit of Kelvin (K). For voltage noise and voltage signals, the NETD is expressed as follows [138]:

(23) $$NETD=\frac{4F^{2}V_{N}}{\tau_{0}A_{D}{ℜ}_{V}{\left(\Delta P/\Delta T\right)}_{\lambda_{1}-\lambda_{2}}}.$$

In terms of current noise and current signals, the NETD can be expressed by the following equation:

(24) $$NETD=\frac{4F^{2}I_{N}}{\tau_{0}A_{D}{ℜ}_{I}{\left(\Delta P/\Delta T\right)}_{\lambda_{1}-\lambda_{2}}}.$$

Here, *F* represents the *F*-number (*F*/#) of the optical system, which is equivalent to the ratio of the focal length *f* to the aperture *D* of the optics [164]. τ_o_ denotes the transmittance of the optics within the wavelength interval ranging from λ_1_ to λ_2_. *A*_D_ stands for the total area of the pixel. (∆P/∆T)_λ1−λ2_ is a black body function that characterizes the change in power per unit area radiated by a blackbody at temperature T with respect to T, as measured from λ_1_ to λ_2_. Specifically, for a 295 K blackbody, the values of (∆P/∆T)_λ1−λ2_ in the 3–5 μm and 8–14 μm spectral intervals are 2.10 × 10^−5^ W/cm^2^ deg K and 2.62 × 10^−4^ W/cm^2^ deg K, respectively [165]. The NETD reflects the sensitivity of radiometers to temperature variations. In order to achieve optimal performance of the bolometer, it is desirable to minimize the NETD value.

#### 2.2.5. Specific Detectivity (D*)

The specific detectivity (D*) proposed is the area-normalized signal-to-noise ratio within the bolometer. It is defined as the signal-to-noise ratio of the pixel output per unit of incident radiant power impinging on the detector, with the measurement being conducted within a 1 Hz bandwidth [166]. The D* reflects the resolving capacity of the bolometer and enables a comparison of the performance of bolometers with different sizes. It can be expressed as follows [138]:

(25) $$D^{*}=\frac{\sqrt{\beta A_{D}\Delta f}}{NEP}$$

Here, *A*_D_ represents the total area of the pixel, *β* denotes the fill factor, ∆*f* indicates the signal detection frequency bandwidth, and *NEP* is as provided by Equation (14). The units of D* are cm·Hz^1/2^/watt. Given that D* is the reciprocal of *NEP*, it is a metric for which a higher value is more favorable. D* can be effectively enhanced either by increasing the responsivity or by reducing the noise. The maximum detectivity achievable for a thermal detector at room temperature while observing a background also at room temperature is approximately 2 × 10^10^ cm·Hz^1/2^/watt, which can be regarded as the theoretical limitation of thermal detectors [167].

## 3. Thermistor Materials for Microbolometers

The thermistor material represents a critical component in microbolometer design, as it fundamentally determines essential device characteristics including the responsivity, sensitivity, noise performance, and overall detection efficiency. To ensure optimal functionality, an ideal thermistor material must satisfy several stringent criteria [133,168,169]: (I) a high temperature coefficient of resistance (TCR), which enables accurate detection of subtle temperature variations; (II) a suitable resistivity that translates into device-level resistance conducive to impedance matching with the readout circuitry, thereby improving the signal fidelity and readout efficiency; (III) low *1/f* noise, which enhances the signal-to-noise ratio and supports the precise measurement of thermally induced changes; and (IV) compatibility with standard CMOS fabrication processes, which facilitates monolithic integration in Si-based platforms. This CMOS compatibility not only streamlines fabrication and reduces production cost but also enables compact system design through direct integration with on-chip electronics, promoting miniaturization and performance scalability. Based on these requirements, a wide range of materials have been investigated for thermistor applications in uncooled microbolometers. Among them, VO_x_, amorphous silicon (α-Si), and titanium oxide (TiO_x_) have achieved broad commercial adoption due to their proven reliability, manufacturability, and performance stability. In parallel, numerous emerging material systems, such as two-dimensional materials, nanocomposites, and engineered oxide heterostructures, are under active investigation and have demonstrated substantial promise for next-generation microbolometers, particularly in enhancing thermal sensitivity and reducing device noise. A comprehensive understanding of these material innovations is essential not only for the advancement of microbolometer performance metrics, such as detectivity, thermal stability, and long-term reliability, but also for the development of novel functionalities that may extend the utility of thermal imaging to new domains. These advancements are instrumental in supporting the continued evolution of microbolometer-based systems across diverse sectors including industrial monitoring, medical diagnostics, and defense-related applications. Ultimately, the integration of high-performance thermistor materials will pave the way for more efficient, robust, and versatile thermal imaging platforms.

### 3.1. Vanadium Oxide Thermistor Material

VO_x_ remains the most widely adopted thermistor material in uncooled microbolometer technologies, owing to its relatively high temperature coefficient of resistance (TCR), typically ranging from −2% to −3%/K, influenced by factors such as oxygen stoichiometry, crystalline phase, and post-deposition annealing conditions [53,145,170,171,172,173,174]. This elevated TCR facilitates substantial resistance modulation in response to minute temperature changes, thereby playing a critical role in the infrared sensitivity of VO_x_-based devices. Despite its commercial maturity, VO_x_ continues to face challenges including long-term thermal stability, reproducibility, and limited compatibility with complementary metal-oxide-semiconductor (CMOS) processes, which constrain its full potential in next-generation infrared systems. As demands for higher sensitivity, reduced noise, and improved integration intensify, enhancing the TCR and overall material properties of VO_x_ has become a focal point of current research. Recent advances have emphasized controlled synthesis methods, precise compositional tuning, and microstructural engineering to not only increase TCR but also maintain the material uniformity, thermal robustness, and process compatibility. These developments in VO_x_ thermistor design are pivotal in extending the performance boundaries of microbolometer systems, thereby enabling more sensitive, stable, and integrable thermal imaging solutions across diverse applications including industrial inspection, biomedical diagnostics, and defense surveillance (Table 1).

As early as 2013, Tsutomu Endoh et al. selected vanadium niobate as a highly sensitive thermistor material, reporting a TCR of −3.6%/K over a broad temperature range, approximately twice that of conventional thermistor materials. This substantial increase in TCR directly enhanced the device’s sensitivity to temperature variations, thereby contributing to improved overall performance [175]. In 2019, Ozer Celik et al. prepared tungsten oxide (WO_3_)-doped vanadium oxide (α-VWO_x_) thin films using reactive DC magnetron sputtering [176]. By incorporating WO_3_ into VO_x_, experimental results revealed that the proportions of V_2_O_5_, VO_2_, and V_2_O_3_ phases varied with changes in the oxygen flow ratio, and WO_3_ doping was found to modulate the oxygen vacancy ratio concentration, thereby influencing the carrier concentration. This alteration in carrier concentration subsequently modified the resistivity. Specifically, the resistivity of α-VWO_x_ increased with a higher oxygen content. Moreover, along with the increase in resistivity, the activation energy of α-VWO_x_ also exhibited a corresponding rise. The combined effect of the rising resistivity and increasing activation energy ultimately results in an improvement in the TCR value. The TCR values of the films prepared at different oxygen flow ratios are 2.45%/K, 2.75%/K, and 3.02%/K, respectively. In addition, amorphous structure suppresses the noise generation at the grain boundaries, reducing the *1/f* noise, which all contribute to the enhancement in device performance. This study demonstrated the potential of WO_3_ doping in improving the TCR values and reducing the noise level of VO_x_ films, making them suitable for microbolometer applications.

**Figure 2:**
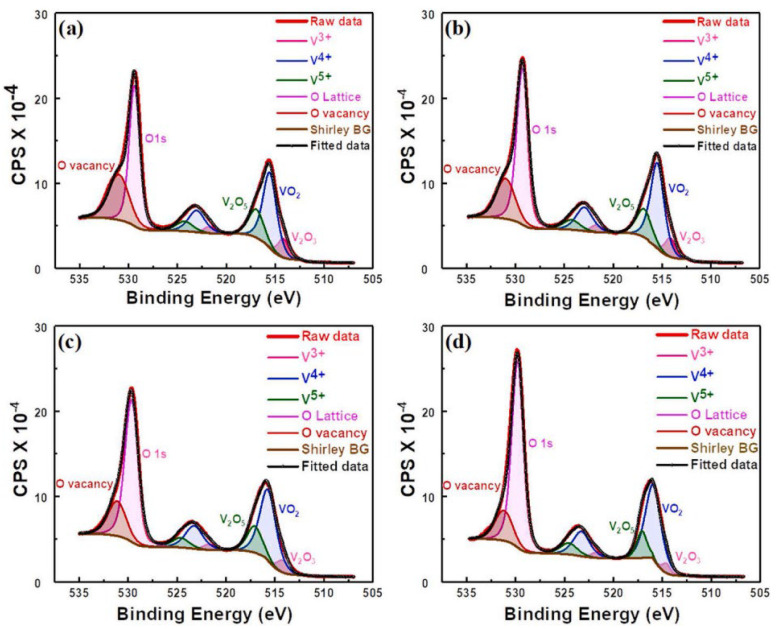
XPS spectra of V2p_3/2_ and O_1s_ in samples with (**a**) 0%, (**b**) 0.74%, (**c**) 2.30%, and (**d**) 4.45% Al loading. Reprinted with permission from ref. [177].

In 2022, Heecheol Shin et al. successfully synthesized the highly uniform Al-doped VO_x_ thin films using atomic layer deposition (ALD) at 80 °C followed by rapid thermal annealing (RTA) at 400 °C for 5 min [177]. RTA treatment was performed for the thermal diffusion of Al dopants and to decrease the resistance of VO_x_ thin films. The introduction of Al dopants suppresses the thermally activated phase transition of the VO_2_ phase, resulting in an increase in TCR up to −4.2%/K and providing enhanced stability over a wide temperature range. X-ray photoelectron spectroscopy (XPS) analysis [180,181,182,183,184,185,186,187] (Figure 2) revealed that Al doping modifies the distribution of oxygen vacancies and alters the valence states of V ions within the film, which also influences the charge transport in Al-doped VO_x_ thin films. However, excessive Al doping reduces the oxygen vacancy concentration and impairs carrier mobility, negatively impacting TCR performance. Specifically, the oxygen vacancy ratio in the film of the undoped Al_2_O_3_ sample was 38%. The oxygen vacancy ratios in the films of the samples doped with Al_2_O_3_ with 1 cycle, 5 cycles, and 10 cycles were 28%, 24%, and 22%, respectively. Correspondingly, as the Al_2_O_3_ cycles increased, the electron mobility of the samples gradually decreased from 6.55 cm^2^/Vs to an undetectable level. In addition, the activation energy also increased from ~a0.34 eV to ~a0.40 eV first and then decreased to ~a0.22 eV. These findings highlight the critical importance of optimizing the Al doping level to optimize the performance improvements in VO_x_ thin films.

In 2023, Usha Philipose et al. deposited the VO_x_ films on Si/SiO_2_ substrates using DC sputter, and then carbon nanotubes (CNTs) with different packing densities were dispersed on the surface of VO_x_ films using a meniscus dragging dispersion (MDD) technique, which was named single-walled carbon nanotube (SWCNT) [178]. The effects of CNT dispersions on the electrical properties and noise signal amplitude of VO_x_ films were extensively studied. For a low-density SWCNT, the TCR value does not change significantly (−3.65%/K–−3.86%/K). Meanwhile, the *1/f* noise is reduced by approximately two orders of magnitude due to the changes in the film surface characteristics (from 6 × 10^−5^ V/√(Hz) to 5 × 10^−7^ V/√(Hz)), which is mainly due to the altered film surface characteristics suppressing the conductivity fluctuations and effectively reducing the noise signal. The conductivity of a high-density SWCNT increases significantly due to more conductive paths having been formed. Meanwhile, the TCR value decreases with an increasing CNT concentration, which was attributed to the chemical interactions between VO_x_ and CNT via oxygen atoms leading to electron transfer and generating more oxygen vacancies in the VO_x_ film. This result indicates that the CNT concentration has an important impact on the electrical properties of the SWCNT film. Therefore, the electrical properties of the SWCNT film can be optimized by reasonably controlling the concentration of CNT.

**Figure 3:**
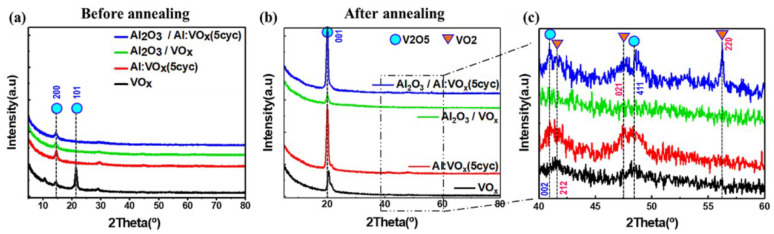
XRD spectra of VO_x_ films before and after PDA: (**a**) before annealing; (**b**) after annealing; (**c**) after annealing from 40–60 degree. Reprinted with permission from ref. [179].

Conventional thermal ALD utilizes oxygen and water for VO_x_ growth, while it utilizes water as the oxidant source for Al_2_O_3_ growth. In contrast, plasma-enhanced atomic layer deposition (PEALD) can utilize oxygen plasma as the main oxidant source for growing both VO_x_ and Al_2_O_3_, which is more effective for Al-doped VO_x_ films. In 2024, Unjeong Kim et al. reported aluminum-doped VO_x_ films fabricated based on PEALD [179]. Oxygen plasma was utilized as the reactant, vanadium tri-isopropoxide (VTOP) as the vanadium precursor, trimethylaluminum (TMA) as the aluminum precursor, and Ar gas as the carrier gas. VO_x_ and Al_2_O_3_ were grown at a low process temperature of 76 °C, and the layer-by-layer deposition mechanism ensured the uniformity of the films. After depositing a 5 nm Al_2_O_3_ passivation layer, the VOx film was subjected to a two-step post-deposition annealing (PDA) treatment (first rapid thermal annealing in N_2_, followed by a 1 h furnace annealing in NH_3_). The X-ray diffractometry (XRD) results showed that the annealing resulted in a significant increase in the crystallinity of the VO_x_ films (Figure 3). Meanwhile, Al dopants effectively reduced the V-O bonds in VO_x_ films and helped to increase the formations of the electro-conducting VO_2_ phase. The strong reducing ability of NH_3_ gas can effectively desorb the oxygen inside the VO_x_ films and sufficiently reduce the VO_x_ films to lower the resistance. Meanwhile Al_2_O_3_ passivation can improve the TCR of VO_x_ film. Al-doped VO_x_ film passivated by Al_2_O_3_ exhibits a TCR value of −3.5%/K at room temperature.

### 3.2. Si and Its Derivatives as Thermistor Materials

Although VO_x_ thermistor materials have been widely utilized in microbolometer applications, they come with a significant drawback of being incompatible with the standard CMOS process technology. To overcome this major hurdle, it becomes crucial to focus on the research and development of CMOS-compatible thermistor materials with a higher TCR value, lower resistivity, and a lower *1/f* noise level, etc. The TCR values of α-Si materials are comparable to those of the VO_x_ thermistor materials (typical values ranging from −2%/K to −3%/K), which can be prepared by PECVD tools, thereby enabling the formation of large-area, low-temperature (<350 °C), and uniformly deposited films and being fully compatible with standard CMOS circuit processes. In addition, α-Si thermistor materials also possess several other advantages, such as good mechanical properties, the ability to form the thin (100 nm) suspended diaphragm with a small thermal time constant of the pixels, low thermal conductivity, higher yield rate, and lower production costs, etc. To explore the material properties of other CMOS compatible materials, pm-Si_x_Ge_1−x_:H, α-Si_x_Ge_y_O_1−x−y_ films, crystalline SiGe/Si QWs, crystalline GeSi/Ge QWs, α-Ge, and α-Ge_1−x_Sn_x_ are also investigated (Table 2).

As early as 2010, pioneers in this field, H. H. Radamson et al., had already carried out a lot of fruitful work in the relevant fields of multiple quantum wells (MQWs) based on SiGe(C) layers [74]. During the manufacturing process, SiGe layers were formed through the interdiffusion of Si into Ge-delta layers that were embedded within Si_0_._99_C_0_._01_ barrier layers, with the growth temperature set at 600 °C. The presence of C atoms within this system played a crucial role in limiting the intermixing of Si and Ge, thereby resulting in the Ge content of the SiGe(C) MQWs reaching as high as 65%. A significant indication of the high quality of the SiGe(C)/Si(C) interface was provided by the presence of a large number of satellites in the high-resolution reciprocal lattice map (HRRLM). Subsequently, detectors with a pixel size of 100 × 100 μm^2^ were fabricated based on these SiGe(C)/Si(C) MQWs. Prior to the metallization of metal contacts, the Ni-silicide step was carried out at 450 °C, and the fabricated device exhibited notable characteristics. It demonstrated a high TCR value of −4.5%/K, as well as an extremely low *1/f* noise constant of 4.4 × 10^−15^. This study effectively demonstrated that carbon doping can not only increase the Ge content within SiGe/Si MQWs but also significantly improve the interface quality between them. Consequently, these MQWs possess fairly high TCR and favorable *1/f* noise characteristics, which make them highly suitable for bolometer applications.

**Figure 4:**
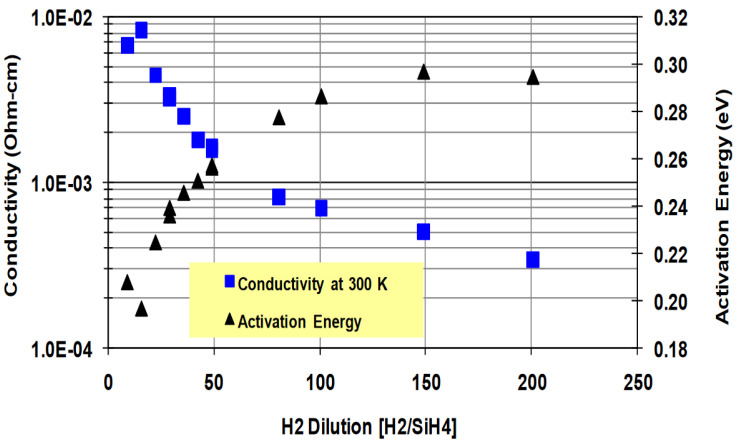
Conductivity and activation energy as a function of hydrogen dilution. Reprinted with permission from ref. [188].

In the same year, Sameer K. Ajmera et al. utilized PECVD with SiH_4_ as the precursor gas to prepare hydrogenated α-Si thin films [188,192]. A detailed investigation was conducted to study the impact of the film growth parameters on the properties of α-Si:H films (specifically hydrogen dilution of SiH_4_ precursors). It was discovered that both the TCR and resistivity of the films increased as the hydrogen dilution (ratio of molecular H_2_ to SiH_4_ in the feed gas of deposition reactor) rose (Figure 4), which was attributed to increased hydrogen dilution during deposition leading to a reduction in the disorder within the amorphous matrix. Furthermore, the effect of doping on the properties of α-Si:H films was also explored in ref [188]. For n-type doping, phosphine (PH_3_) was used, while for p-type doping, either B_2_H_6_ or BCl_3_ was employed. The results indicated that for both n-type and p-type doping, the film’s resistivity was reduced with the growth of the dopant concentration in the deposited plasma. Moreover, saturation behavior occurred when the dopant gas flow was higher. The resistivity conformed to the Arrhenius thermally activated dependence whenever the temperature was at room temperature or higher. As the temperature decreased, the conductivity transitioned from Arrhenius behavior to a variable range jump mechanism described by the Mott relation. The prepared n-type and p-type α-Si films exhibited a wide resistivity range, from approximately 200 Ω·cm to around 1.0 × 10^5^ Ω·cm at room temperature, with the corresponding TCR ranging from −2.5%/K to approximately −5%/K. Generally, n-type doped films demonstrated higher TCR than p-type doped films of the same resistivity due to the different Arrhenius prefactors. Through meticulous selection of deposition parameters, the TCR of α-Si thin film was enhanced from a baseline of −2.5%/K to approximately −3.9%/K. Consequently, the electrical properties of the α-Si film can be optimized by controlling the hydrogen dilution and doping level. This optimization is expected to improve the pixel responsivity without increasing the thermal time constant.

In 2018, M. Lutful Hai et al. prepared the α-Si_x_GeyO_1−x−y_ films for uncooled infrared microbolometers through the RF magnetron sputtering technique. In this process, they simultaneously deposited two separate targets (Si and Ge targets are both used) within a mixed environment of argon and oxygen [189]. The experimental results demonstrated that the TCR and resistivity of α-Si_x_GeyO_1−x−y_ films are influenced by the proportions of Si, Ge, and oxygen within the film, which can be regulated by the sputter deposition process. By optimizing the composition to achieve a relatively low oxygen concentration (7–8%), it is possible to increase the TCR and decrease the resistance. For instance, α-Si_0_._054_Ge_0_._877_O_0_._069_ films deposited at room temperature exhibit a relatively high TCR of −3.5 %/K and an acceptable resistivity of 629 Ω·cm.

**Figure 5:**
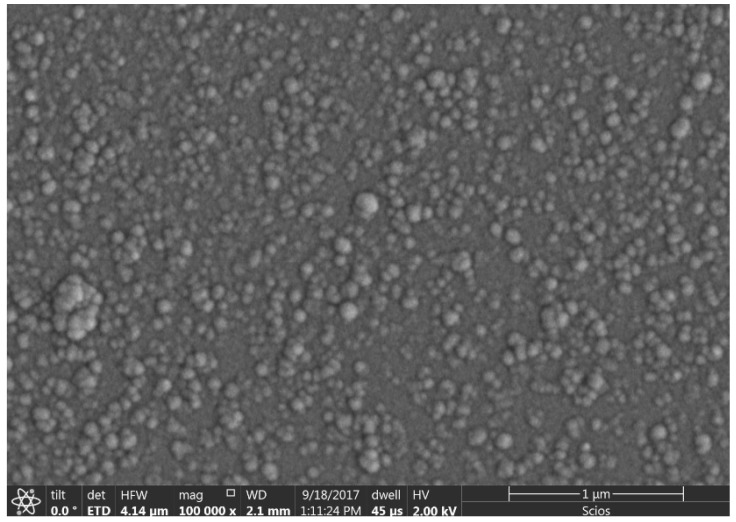
FE-SEM image of the pm-Si_x_Ge_1−x_:H surface [190]. Open access by MDPI, 2020.

In 2020, Ricardo Jimenez et al. carried out the deposition pm-Si_x_Ge_1−x_:H) at a temperature of 200 °C by employing PECVD [190]. It is clear from the high-resolution field emission scanning electron microscopy (FE-SEM) results that several Si-Ge nanoclusters on the film can be observed (as shown in Figure 5). The pm-Si_x_Ge_1−x_:H thermistor film was found to possess a TCR of −4.08%/K and a conductivity value of 1.5 × 10^−5^ S·cm^−1^.

**Figure 6:**
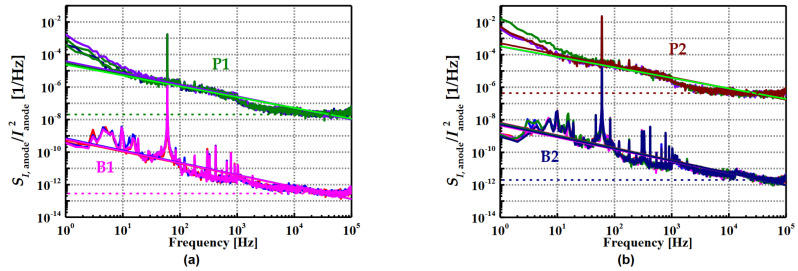
*1/f* noise measurement results for (**a**) high SCCM types and (**b**) low SCCM types. Reprinted with permission from ref. [191].

In 2023, Ki-Nam Kim et al. conducted an investigation into the effects of doping on α-Si:H thin films [191]; four types of α-Si:H films were formed by modulating the dopant gas flow in the PECVD tools, including high standard cubic centimeters per minute (SCCM) boron-doped, low SCCM boron-doped, high SCCM phosphorus-doped, and low SCCM phosphorus-doped films. In contrast to conventional Si devices where the mobility of n-types is typically 2–4 times higher than that of p-types, n-type α-Si:H films exhibited higher resistance than p-type α-Si:H films; this tendency was also reflected in both the thin-layer resistance and resistivity. Specifically, the resistivity of the high SCCM phosphorus-doped and low SCCM phosphorus-doped films was measured to be 1.49 × 10^5^ Ω·cm and 2.48 × 10^6^ Ω·cm, respectively. Meanwhile, the resistivity of the high SCCM boron-doped and low SCCM boron-doped films was 2.28 × 10^3^ Ω·cm and 3.45 × 10^3^ Ω·cm, respectively. Moreover, it was found that n-type α-Si:H films with higher resistance had higher TCR values compared to p-type α-Si:H films. The TCR values of the high SCCM phosphorus-doped and low SCCM phosphorus-doped films were −5.01%/K and −5.08%/K, respectively. On the other hand, the TCR values of the high SCCM boron-doped and low SCCM boron-doped films were −3.01%/K and −4.16%/K, respectively. The reason for the decrease in the TCR value with an increase in dopant concentration lies in the fact that the increase in the extended state in the grain boundary occurs at a slower rate than the increase in dopant concentration. In addition, it was noted that the temperature dependence of the n-type α-Si:H films was more pronounced. The *1/f* noise was measured (Figure 6). The results show that the magnitude of the *1/f* noise also decreases with increasing doping concentration, which is due to the fact that the number of trap states increases with decreasing dopant gas. Interestingly, although the TCR of p-type a-Si:H is not as good as that of n-type a-Si:H, the *1/f* noise characteristics are better. Overall, these findings clearly demonstrate that the doping type and concentration have a significant impact on the properties of α-Si:H films.

### 3.3. TiO_x_ Thermistor Materials

Similar to vanadium, titanium is capable of forming a variety of oxides, encompassing both conductive and insulating phases. Titanium oxide (TiO_x_) can exist in multiple crystalline or amorphous states, with its physical properties highly dependent on specific film deposition conditions. Owing to its excellent thermal stability, suitability for large-area fabrication, and compatibility with standard CMOS processes, TiO_x_ has attracted considerable attention and has been extensively investigated as a thermistor material. In recent years, TiO_x_ has also been adopted in commercial microbolometer applications, establishing itself as the next widely implemented metal oxide thermistor material following VO_x_ (Table 3).

In 2008, Myung-Ho Kwon et al. fabricated TiO_2−δ_ thin films by reactive sputtering, the resistivity of which ranged from 10^−2^ Ω·cm to 10 Ω·cm. The TiO_2−δ_ thin film with a resistivity higher than 1 Ω·cm featured the maximum TCR value of −2.8 %/K. Unfortunately, this TCR value is difficult to stably reproduce due to the metastability of the TiO_2−δ_ film deposition process. Finally, the TiO_2−δ_ film with −2.1 %/K TCR and 1 Ω·cm resistivity was used as the thermosensing material for the microbolometer due to its suitable TCR value, resistance, and predominant stable deposition condition. Moreover, this TiO_2−δ_ thin film also exhibits a *1/f* noise constant (6.2 × 10^−14^) comparable to VO_x_. The thermal response time and NETD of the 50 μm × 50 μm microbolometer are 7.5 msec and 34 mK, respectively [193]. Since then, the i3 system Company has been dedicated to TiO_x_-based uncooled FPA. Recently, 1280 × 1024 TiO_x_-based FPA with a pixel size of 8 μm has been reported. The 8 μm pixel structure no longer adopts a simple single-level design; instead, it employs an umbrella structure produced by 8-inch based 0.18 μm 3D MEMS process technology to enhance the fill factor. The readout integrated circuit is also manufactured with the 0.18 μm CMOS technology, which utilizes a four-channel integrated structure to minimize the thermal noise resulting from the resistivity of TiO_x_ film. The NETD and thermal response time of the 1280 × 1024 TiO_x_-based FPA uncooled 8 μm FPA are 45 mK and 11 msec, respectively [198].

**Figure 7:**
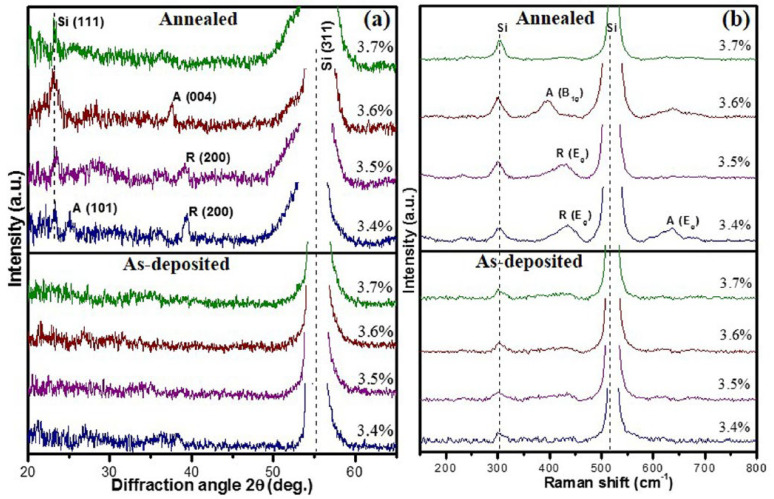
XRD and Raman of as-deposited and annealed TiO_2−x_ films at various R_O2_. (**a**) XRD spectra for as deposited and annealed sample; (**b**) Raman spectra for as deposited and annealed sample. Reprinted with permission from ref. [194].

In 2015, Y. Ashok Kumar Reddy et al. prepared TiO_2−x_ thin films on Si/SiO_2_ substrates using RF reactive magnetron sputtering. Oxygen and argon were used as the reactive and sputter gases, respectively [194]. TiO_2−x_ films were deposited at different relative mass flow of oxygen (R_O2_) levels (3.4–3.7%) and thermally annealed at 300 °C for 1 h in an air environment. X-ray diffraction and Raman results (Figure 7) show that the annealing caused the TiO_2−x_ film structure to change from amorphous to crystalline (rutile/anatase). This is attributable to oxygen deficiency, which can be offset with increased R_O2_, and the TCR also increased from 2.54% to 3.65%. TiO_2−x_ films with higher R_O2_ levels show higher resistivity, lower carrier density, and higher activation energy. In addition, annealing has been shown to effectively improve the *1/f* noise parameter but will slightly reduce the TCR value.

**Figure 8:**
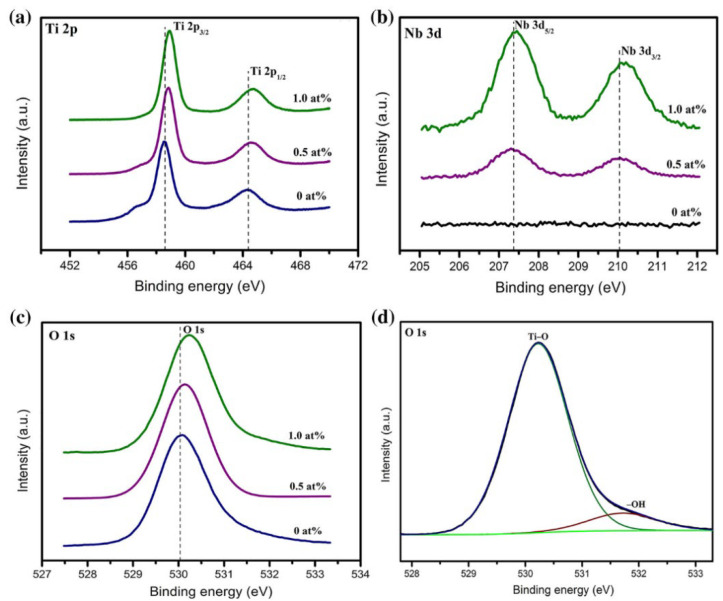
The XPS spectra of Nb:TiO_2−x_ films at different Nb concentrations: (**a**) Ti2p; (**b**) Nb3d; (**c**) O1s with the composition of 0%, 0.5%, and 1.0%; (**d**) O1s for Ti-O and OH. Reprinted with permission from ref. [196].

In 2016, Mahmud Yusuf Tanrikulu et al. investigated the effect of the annealing temperature on the properties of TiO_x_ thin films grown by the atomic layer deposition (ALD) technique [195]. The XPS results indicate that the deposited films are amorphous. As diffusing oxygen fills the vacancies, the O:Ti ratio of TiO_x_ films increases with the temperature and reaches a maximum at 475 °C. When the annealing temperature exceeds 475 °C, the O:Ti ratio decreases slightly because of the anatase-to-rutile phase transition. The TiO_x_ film grown at 150 °C and annealed at 300 °C has a surprisingly high TCR value of −7.2%/K at room temperature. In 2018, Y. Ashok Kumar Reddy et al. investigated the effects of the Nb-doping concentration on the bolometric properties of TiO_2−x_ films [196]. Three doping concentrations of TiO_2−x_ films were deposited at room temperature by radio frequency reactive sputtering using 0 at. % (pure titanium), 0.5 at. %, and 1 at. % Nb-doped Ti metal targets, respectively. The XPS results confirm that the Nb concentrations of the TiO_2−x_ films are the same as the respective targets, 0 at. %, 0.5 at. %, and 1 at. %, respectively (Figure 8). For the 0 at. %, 0.5 at. %, and 1.0 at. % Nb-doped TiO_2−x_ films, the relative mass flow rate of oxygen was adjusted to 3.4%, 3.9%, and 4.5% respectively, with the aim of achieving a similar resistivity for TiO_2−x_ films. The resistivities of 0 at. %, 0.5 at. %, and 1.0 at. % Nb-doped TiO_2−x_ films were 0.82 Ω·cm, 0.75 Ω·cm, and 0.69 Ω·cm respectively. The activation energy of TiO_2−x_ films and their corresponding TCR values increased with the increase in the Nb-doping concentration. The TCR values of the 0 at. %, 0.5 at. %, and 1.0 at. % Nb–doped TiO_2−x_ films were −2.54%/K, −2.65%/K, and −2.78%/K, respectively.

**Figure 9:**
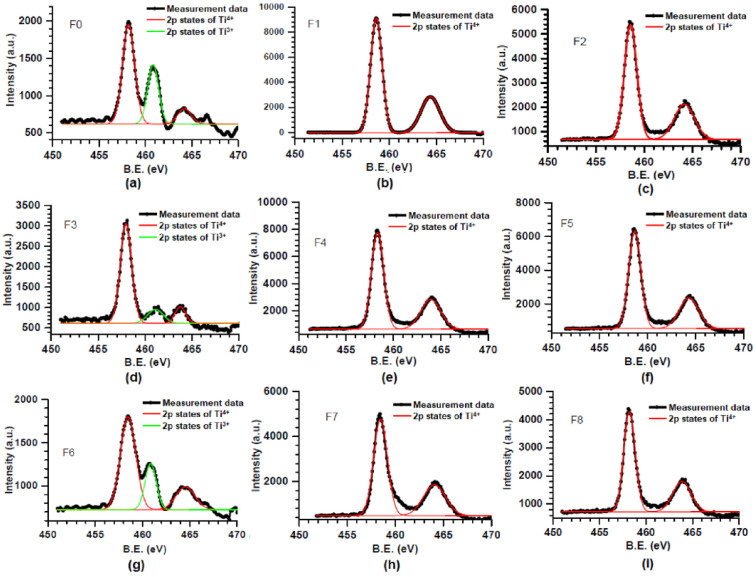
Core level Ti_2p_ spectra for the deposited films at different oxygen flow rates: (**a**) F0; (**b**) F1; (**c**) F2; (**d**) F3; (**e**) F4; (**f**) F5; (**g**) F6; (**h**) F7; (**i**) F8. Reprinted with permission from ref. [197].

In 2022, Isha Yadav et al. investigated the effects of the thickness and stoichiometry of TiO_x_ films on their electrical and thermal properties [197]. The TiO_x_ films were deposited by RF reactive magnetron sputtering at room temperature onto Si substrates (with 100 nm SiO_2_) and glass substrates with prefabricated electrodes. The stoichiometry of the TiO_x_ films was controlled by varying the oxygen flow rate. The XPS analysis revealed that only Ti^4+^ and Ti^3+^ states were present in the deposited films, with no Ti^2+^ states detected. The compositions of the prepared TiO_x_ films were x = 1.5, x = 1.7, and x = 1.9, respectively (Figure 9). The measurement results indicate that the electrical properties of TiO_x_ films are closely related to their stoichiometry. As the “x” values increase, the TCR values of TiO_x_ films rise from approximately −1.12%/K to −4.87%/K, the activation energy increases from 0.099 eV to 0.48 eV, and the estimated room temperature resistivities increase from 1.2 Ω·cm to 58.7 Ω·cm. Since oxygen vacancies can act as charge carriers, the conductivity of TiO_x_ films increases with the number of oxygen vacancies. Non-stoichiometric TiO_x_ films with higher “x” values have fewer oxygen vacancies than those with lower x-values, thus resulting in an increase in TCR, room temperature resistivity, and activation energy. The cross-plane thermal conductivities of non-stoichiometric TiO_x_ films were estimated using the 3ω technique. The results show that thermal conductivities of non-stoichiometric TiO_x_ films are independent of the composition and positively correlated with the film thickness. The thermal conductivities of the TiO_x_ films with thicknesses of 100, 200, and 400 nm were measured to be approximately 0.5, 1.2, and 2.4 W/mK, respectively. This is mainly because thermal conductivity of semiconductor materials is mainly affected by phonons, and the phonon scattering decreases as the film thickness increases.

### 3.4. Other Thermistor Materials

VO_x_ and α-Si have long been the most commonly utilized thermistor materials in commercial FPA microbolometers. More recently, microbolometers incorporating TiO_x_ as the thermistor material have also been introduced into the commercial market. Despite these advancements, the search for novel thermistor materials that offer a high TCR, suitable resistivity, and superior noise characteristics remains an active area of research. Several emerging materials have already demonstrated promising bolometric performance and are considered strong candidates to potentially replace conventional thermistor materials in future commercial microbolometer applications (Table 4).

In 2003, A. Torres et al. prepared amorphous germanium (α-Ge:F) thin films using a mixture of GeF_4_ and H_2_ by low-frequency PECVD at 300 °C [199]. At room temperature, the 0.1 μm thick α-Ge film exhibits a higher TCR value of −5%/K and a moderate conductivity of 2.5 × 10^−3^ Ω^−1^ cm^−1^.

**Figure 10:**
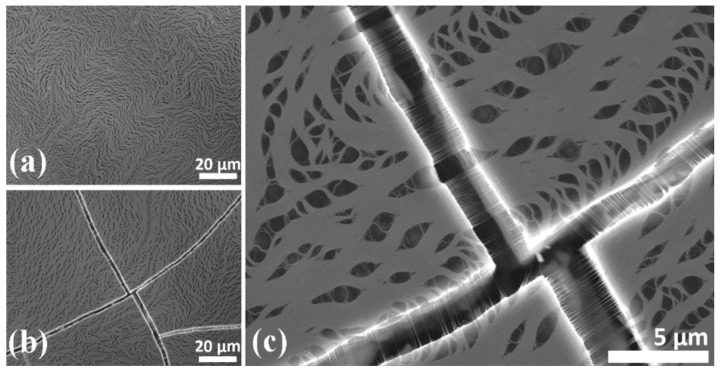
SEM images of (**a**) an uncracked film and (**b**) cracked film, with (**c**) higher magnification. Reprinted with permission from ref. [200].

In 2015, Trevor J. Simmons et al. prepared single-walled carbon nanotube (SWCNT) composite films. They dropped a sonicated mixture, which consisted of SWCNTs, surfactant sodium dodecylbenzenesulfonate, and polyvinylpyrrolidone, onto a polished silica substrate and then dried it horizontally for several hours [200]. The authors initially investigated how the morphology of the SWCNT composite affected the bolometer performance. It was discovered that SWCNT composite films dried rapidly in an environment with a relative humidity of around 15% would randomly form micron-scale cracks (Figure 10a). These films had a TCR value of −3.8%/K ± 1.2%/K. In contrast, SWCNT composite films that dried slowly at a relative humidity of approximately 80% were very uniform and had few cracks (Figure 10b). These films had a TCR value of −6.5%/K ± 2.9%/K. The responsivity of the cracked and uncracked films was 62.5 V/W and 51.0 V/W, respectively, at a bias voltage of 500 mV. The higher responsivity of the cracked films might be attributed to SWCNTs that were suspended across the cracks on the substrate, thus achieving higher thermal isolation (Figure 10c).

In addition, the effect of the chirality of the SWCNT composite films on the bolometric characteristics was also investigated. Composite films containing semiconductive, metallic, and a 1:1 mixture of these two types of SWCNTs were deposited in a humid environment to prevent cracking, because the cracks in cracked films occur randomly, and the key figure of merit is lower than uncracked films. The conductivities of the composite films containing semiconductive, metallic, and SWCNTs were 0.9 × 10^−8^ S m^−1^, 2.1 × 10^−8^ S m^−1^, and 1.9 × 10^−8^ S m^−1^, with TCR values of −6.5%/K ± 1.9%/K, −2.3%/K ± 0.9%/K, and −3.0%/K ± 0.4%/K, respectively. The enriched chirality semiconductive SWCNTs exhibited the largest TCR values and the highest variability. On the other hand, a mixture of metallic and semiconductive SWCNTs also exhibited moderately improved TCR values and the lowest variability, proving to be potentially suitable for high-performance bolometer applications.

**Figure 11:**
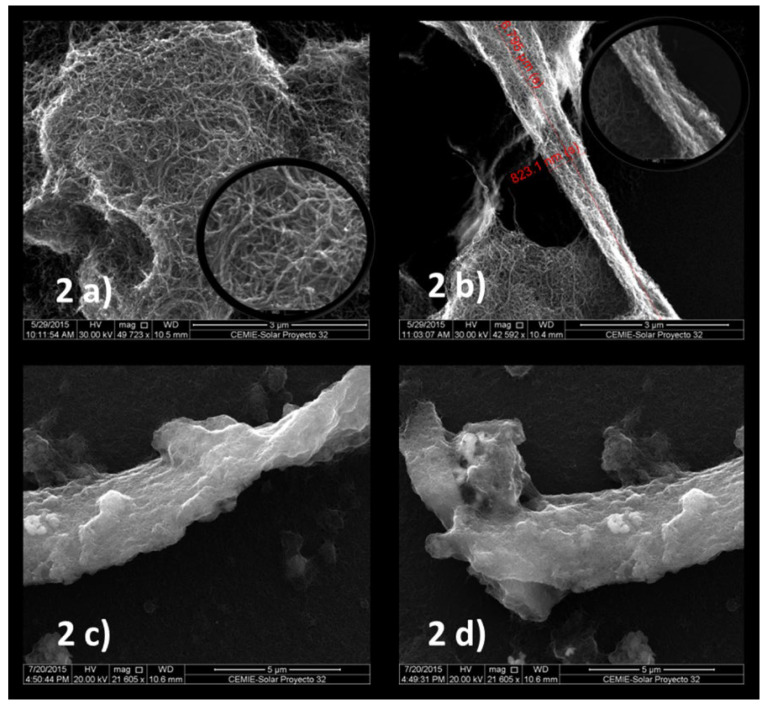
SEM images of (**a**) pristine MWNT and (**b**–**d**) t-MWNT deposited on Si. Reprinted with permission from ref. [201].

In 2017, Guadalupe García-Valdivieso et al. successfully prepared thymine-functionalized multi-walled carbon nanotube (t-MWNT) thin films [201]. Thymine molecules were covalently attached to MWNT, and the resulting product was dispersed in dimethylformamide at a 1:4 ratio. MWNT films were fabricated by drying a carbon nanotube solution, which was deposited onto a polished Si substrate. The thickness of the film was determined by the number of solution drops. As can be observed from the SEM images, pristine MWNTs display the typical spaghetti-like entanglement of individual nanotubes (Figure 11a). In contrast, t-MWNTs clearly exhibit parallel arrays of thick braided and entangled CNTs (Figure 11b–d). The electrical test results indicate that the average TCR value of the thymine-functionalized MWNTs films, with a dried layer thickness of 0.53 ± 0.05 μm, is −5.6 ± 0.1%/K, at temperatures ranging from 297 K to 308 K.

It should be noted that carbon nanotube films are typically prepared via wet processes rather than traditional thin film deposition techniques. Surprisingly, this preparation process is fully compatible with current mainstream microbolometer manufacturing processes. Recently, the NEC Corporation demonstrated a 640 × 480 pixel bolometer-type uncooled IRFPA fabricated using SWCNTs. The detailed process will be described in Section 4.3.

**Figure 12:**
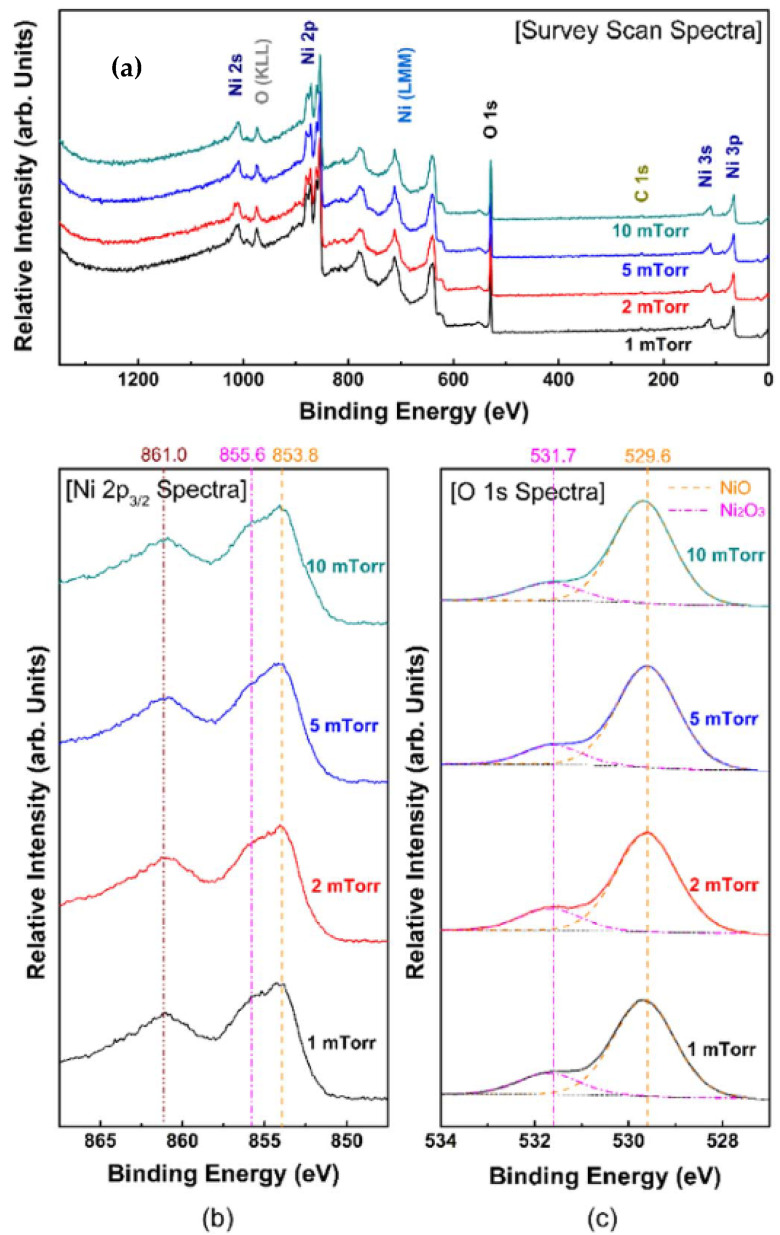
XPS spectra of Ni_1−x_O films deposited at various sputtering pressures: (**a**) survey scan spectra; (**b**) Ni 2P_3/2_ spectra; (**c**) O1s spectra. Reprinted with permission from ref. [202].

In 2017, In–Ku Kang et al. prepared polycrystalline Ni_1−x_O thin films by using reactive sputtering and investigated the effect of the sputtering pressure on the film properties [202]. When the sputtering pressure is increased from 1 mTorr to 10 mTorr, atoms experience more collisions before reaching the substrate, which leads to a decrease in their kinetic energy and limited diffusion. As a consequence, both the lattice constant and crystal size increase with the sputtering pressure. The XPS analysis revealed that the deposited Ni_1−x_O films consisted of Ni ions with chemical bonding states of +2 and +3 (Figure 12). Higher sputtering pressures resulted in better stoichiometry and fewer structural defects in the Ni_1−x_O films. The TCR values of Ni_1_−_x_O films deposited at sputtering pressures of 1 mTorr, 2 mTorr, 5 mTorr, and 10 mTorr were −2.76%/K, −2.82%/K, −3.80%/K, and −5.62%/K, respectively. The corresponding conductivities were 9.78 × 10^−2^ S/cm, 7.49 × 10^−2^ S/cm, 4.87 × 10^−3^ S/cm, and 2.20 × 10^−5^ S/cm, respectively. On the other hand, the *1/f* noise increases with the sputtering pressure. Since the TCR and *1/f* noise have opposite effects on the bolometric performance, (αH/n)^1/2^/|β|, a material figure of merit that determines the minimum achievable noise equivalent temperature (NET), is introduced to further evaluate the bolometric performance of Ni_1−x_O films, which confirmed that Ni_1−x_O films deposited at lower sputtering pressures perform better in bolometer applications.

**Figure 13:**
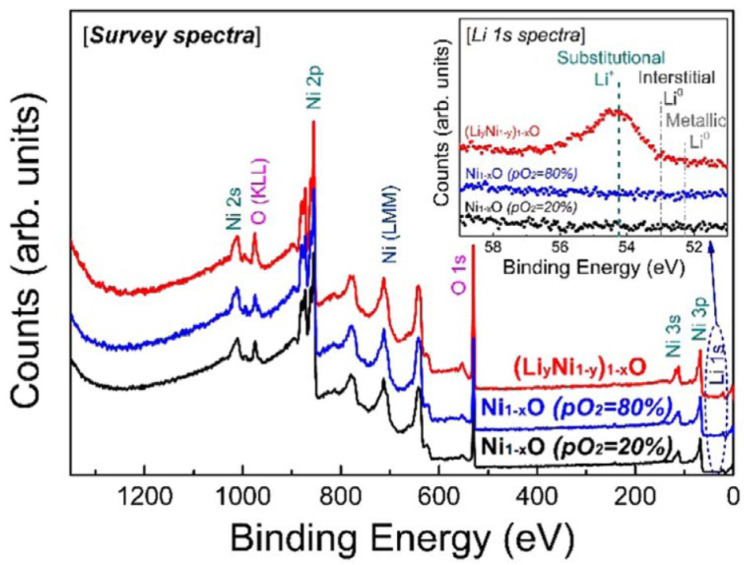
XPS spectra of (Li_y_Ni_1−y_) _1−x_O film and Ni_1−x_O films deposited at various sputtering pressures. Reprinted with permission from ref. [203].

To improve the sensing properties of NiO_x_ films, authors also investigated the lithium (Li) incorporation effect [203]. (Li_y_Ni_1−y_)_1−x_O films were deposited by RF magnetron sputtering using a Li_0_._2_Ni_0_._8_O target. The XPS results show that Li ions only occupy Ni sites and do not create an interstitial Li defect (Figure 13). The substitutional incorporation of Li led to an increase in the number of hole carriers in the (Li_y_Ni_1−y_)_1−x_O film. Compared with the Ni_1−x_O film deposited at an oxygen fraction of 20% with similar crystallinity and structural defects, the (Li_y_Ni_1−y_)_1−x_O film has higher electrical conductivity and smaller contact resistance. In addition, the (Li_y_Ni_1−y_)_1−x_O film exhibits a (αH/n)^1^/^2^/|β| value that is 10 times smaller than that of the Ni_1−x_O film, which is mainly due to the (Li_y_Ni_1−y_)_1−x_O film generating substantially lower *1/f* noise than the Ni_1−x_O film while maintaining a higher absolute TCR value of −2.78%/K.

**Figure 14:**
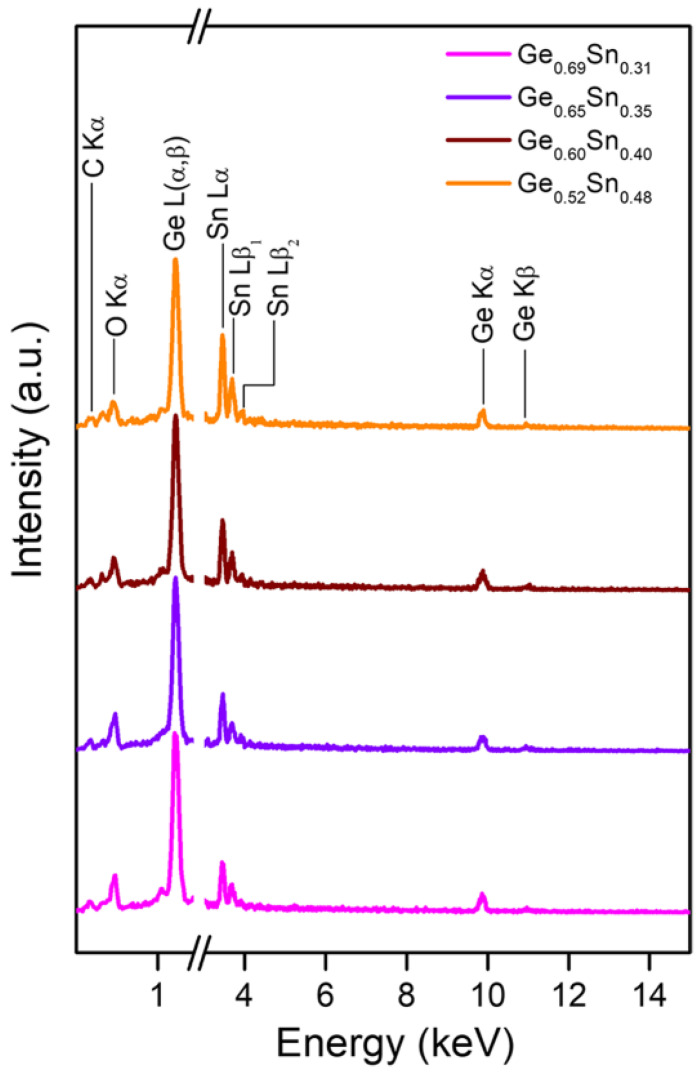
EDX spectra of the Ge_1−x_Sn_x_ films deposited at various DC powers. Reprinted with permission from ref. [204].

Generally, there are several types of tools that can be used for the growth of germanium–tin (Ge_1−x_Sn_x_) alloys, such as molecular beam epitaxy (MBE) [205,206,207], CVD [208,209,210,211], physical vapor deposition (PVD), and sputtering [212,213], etc. The deposition temperature can be controlled below 350°C, ensuring thermal compatibility with back-end-of-line (BEOL) processes in Si ROICs. While this low-temperature capability is advantageous, we note that high Sn-content films can introduce lattice mismatch and strain relaxation, potentially affecting the film uniformity and long-term stability-issues that require further optimization for large-scale manufacturing. From a scalability perspective, Esam Bahaidra et al. synthesized Ge_1−x_Sn_x_ thin films with a thickness of 200 nm at room temperature through a co-sputtering process on Si substrates with 300 nm SiO_2_ [204], and the effect of Sn concentration on the properties of Ge_1−x_Sn_x_ thin films was further investigated. From the energy-dispersive X-ray (EDX) results, the Sn concentration in the deposited Ge_1−x_Sn_x_ thin films is 31%, 35%, 40%, and 48% for a Sn deposition DC power of 30, 50, 70, and 100 W, respectively (Figure 14). The sheet resistance of the Ge_1−x_Sn_x_ films diminishes with the augmentation in the Sn concentration on account of the elevated electron concentration within the conduction band. If the Sn concentration fulfills the range of 0.4 < x ≤ 0.48, the Ge_1−x_Sn_x_ films will undergo a transition from semiconductor behavior to metallic behavior. Thus, the resistivity of the Ge_1−x_Sn_x_ films exhibits an exponential decrease with the increasing Sn concentration. Specifically, when the Sn concentration escalates from 17% to 40%, the resistivity of the Ge_1−x_Sn_x_ films diminishes from 164.6 Ω·cm to 4.86 Ω·cm. In contrast, the TCR value decreases linearly with an increasing Sn concentration percentage (−3.96%/K to −2.52%/K). In addition, microbolometers with pixel areas of 10 × 10 μm^2^ without an air gap were fabricated using Ge_0_._69_Sn_0_._31_ thin films thermistor materials, and their electro-optical performance was also investigated. The fabricated microbolometer exhibited a signal-to-noise ratio of 364 Hz^1/2^ and a voltage responsivity of 0.11 V/W at a modulation frequency of 200 Hz. Meanwhile, an NEP of 1.2 mW/√Hz was obtained. For a microbolometer with an air-bridge configuration, having the same pixel area and under the assumption of the same noise density, the anticipated voltage responsivity and NEP are 28.5 × 10^3^ V/W and 4.6 × 10^−6^ mW/√Hz, respectively. Regarding the integration strategies, both direct deposition onto completed ROIC wafers (enabled by the low thermal budget) and wafer bonding approaches could be pursued, depending on the specific ROIC metallization scheme. For SWCNT networks, scalable solution-based deposition and transfer-printing methods are being actively explored for large-area integration, and recent advances have demonstrated uniform arrays on wafer-scale substrates with resistivity and TCR suitable for uncooled IR sensing. These developments provide a promising foundation for future heterogeneous integration with CMOS ROICs.

**Figure 15:**
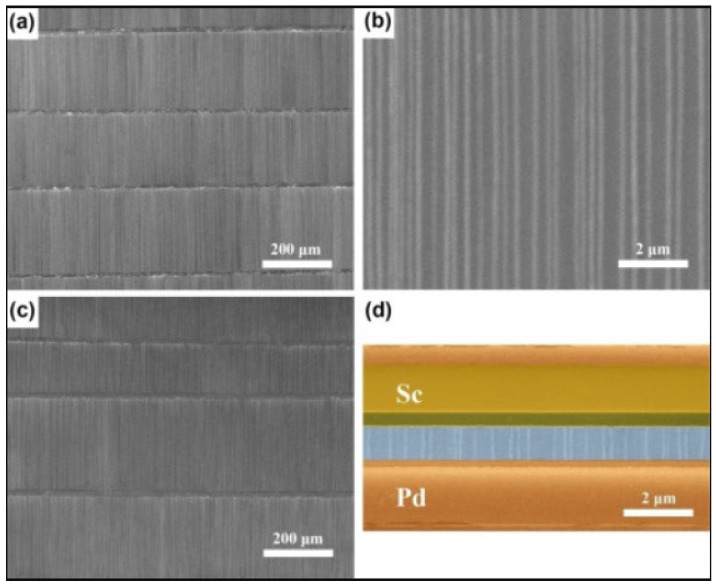
Fabrication of the asymmetrically contacted CNT thin film diode. (**a**) Low and (**b**) high magnification SEM images of the aligned SWCNTs grown on quartz. (**c**) Arrays of SWCNTs on Si/SiO_2_ substrate after being transferred from quartz. (**d**) Typical SEM image of thin film diode contacted with Sc and Pd as source and drain, respectively, with channel width W = 20 μm and channel length L = 1 μm. Open access by Optica Publisher, 2012.

Later, T. Tanaka et al. extracted the semiconducting SWCNTs with nonionic surfactants by using the electrical-field inducing layer formation (ELF) method [214]. By applying a voltage to the mixed solution, the semiconducting and metallic SWCNTs will separate toward the anode and cathode, respectively. After several tens of hours, the semiconducting SWCNTs with purity higher than 98% were extracted. Atomic force microscopy (AFM) images showed that the SWCNTs were densely fixed on SiO_2_ with 3-Aminopropyltriethoxysilane (APTES) self-assembled monolayers formed on the surface and oriented networks in local regions (Figure 15). The TCR value of the SWCNT networks is close to −6%/K at room temperature. A year later, the authors demonstrated bolometer-type uncooled infrared focal plane allays (IRFPAs) of 640 × 480 pixels fabricated using high-purity SWCNTs. The responsivity and thermal time constant of a typical element in the LWIR region exceed 105 (*v*/*w*) @ 0.2 V and 5 ms, respectively. The great potential of semiconducting SWCNTs for realizing highly sensitive thermal imaging is demonstrated.

## 4. Microbolometer Physical Design and Manufacturing

In the contemporary technological landscape, infrared imaging has assumed an increasingly critical role, with wide-ranging applications across diverse fields. Microbolometer IRFPAs, as the fundamental components of uncooled infrared imaging systems, are pivotal to the advancement of infrared imaging technology. Enhancing their performance is essential for the continued progress of the field. Research on microbolometer IRFPA technologies holds significant importance. From a technological development perspective, optimizing the thermal isolation structures and optical designs effectively enhances the sensitivity and resolution of microbolometers.

### 4.1. Thermal Isolation Structure and Optical Design

**Figure 16:**
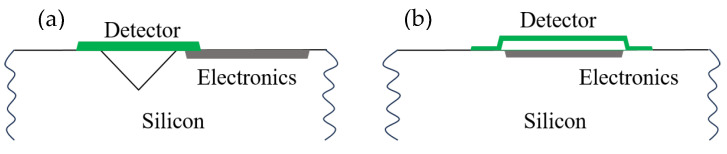
Cross section of pellicle-supported structure (**a**) and bridge-supported structure (**b**) of a microbolometer [145].

Realizing a high-performance microbolometer necessitates careful consideration of multiple design parameters and inherent trade-offs. Key factors influencing device performance include the thermal conductance to the surroundings, infrared absorption efficiency, pixel fill factor, TCR, and the *1/f* noise characteristics of the thermistor material. Among these, thermal conductance is widely recognized as the most critical parameter, as established by thermal detector theory [215]. In microbolometer IRFPAs, heat transfer occurs via conduction, convection, and radiation. However, in evacuated packages, commonly employed to enhance sensitivity, the convection and radiation are negligible, rendering conductive heat transfer from the active sensing region to the substrate the dominant mechanism. The thermal isolation structure plays a pivotal role in defining this thermal conductance, and variations in its design can lead to substantial performance differences. These isolation structures must not only provide effective thermal insulation but also be mechanically robust, compact, and compatible with scalable manufacturing processes. Microelectromechanical systems (MEMS) technology has enabled the practical realization of such structures by facilitating the selective etching of materials and crystalline planes to form thermally insulated microstructures [145,216,217,218,219]. Contemporary microbolometer designs commonly employ pellicle-supported or bridge-supported configurations to minimize the thermal conductivity (Figure 16).

Microbolometer structures can be mainly divided into two types: pellicle-supported structures [220,221,222] and bridge-supported structures [223,224,225]. For the pellicle-supported structure of microbolometers (Figure 16a), surface micromachining and bulk micromachining are combined for the fabrication process [226]. The detector element is formed on a pellicle that is approximately level with the Si surface. Through bulk micromachining, a cavity is etched in the underlying bulk Si to thermally isolate the detector element from the substrate. This cavity can be anisotropically etched from either the front or back side of the Si substrate. Since the Si beneath the detector element is partially removed by etching, readout electronics can only be positioned adjacent to the sensitive area, which results in a relatively limited fill factor of around 20% [145,227,228].

Although pellicle-supported microbolometer structures have been explored, the bridge-supported configuration has emerged as the predominant architecture in current technologies (Figure 16b). Advances in micromachining techniques have enabled the fabrication of bridge-supported microbolometers entirely through surface micromachining processes, wherein the detector element is suspended above the silicon substrate and thermally and electrically connected via two long narrow support legs and studs. This architecture offers several key advantages. Notably, it preserves the integrity of the silicon substrate, allowing readout electronics to be integrated directly beneath the suspended microbridge. As a result, the bridge-supported structure achieves a significantly higher fill factor compared to pellicle-supported designs. Furthermore, it provides superior thermal insulation, which is critical for enhancing sensitivity and enables precise control over the resonant cavity length. Collectively, these benefits have established the bridge-supported structure as the dominant design paradigm in modern resistive microbolometer technologies.

**Figure 17:**
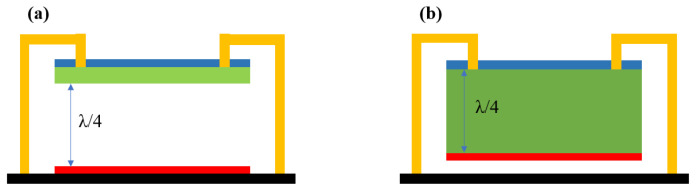
(**a**) The optical quarter-wave resonant cavity is formed in a vacuum, with the reflective layer positioned on top of the readout circuit. (**b**) The optical quarter-wave resonant cavity is formed by the thermistor material itself, with the reflective layer located at the bottom of the thermistor film.

Infrared radiation absorption is achieved through a synergistic combination of an infrared absorbing film, a metal reflector deposited on the substrate, and the cavity between the infrared absorbing film and metal reflector. The infrared absorbing film, placed on top, converts the incident infrared radiation it absorbs into heat and then transfers this heat to thermistor film [229,230]. The infrared absorbing film must possess high absorption efficiency within the operating wavelength range and be compatible with conventional post-CMOS Si micromachining technologies. The SiN_x_ passivation layer is commonly used as an absorbing layer for infrared radiation in commercial bolometers, with an absorption peak around 11.5 μm [226]. Metal film is another viable option; it not only absorbs infrared radiation but also functions as an electrical contact. By adjusting the thickness of the metal film such that its impedance approaches the free-space impedance (377 Ω/sq), the absorption of infrared radiation can be maximized [231,232]. At ambient temperature, the electromagnetic blackbody radiation emitted by objects has a maximum intensity at a wavelength of approximately 10 μm [73]. Therefore, the distance between suspended film and metal reflective layer is set to around 2.5 μm (a quarter of the wavelength of target incident infrared radiation) to form an optical resonant cavity (Figure 17a). The quarter-wave optical cavity enables the infrared radiation passing through the absorbing layer to be trapped within the cavity and reabsorbed by thermistor film, thus enhancing the absorption of incident infrared radiation. An alternative approach is to utilize the thermistor material itself, which is a quarter-wave thick of target incident infrared radiation, to act as an optical resonance cavity. In this instance, Figure 17b shows that the reflective layer is positioned on the bottom of the thermistor material [167].

### 4.2. Fabrication Process Flow

In the field of microbolometer technology, two primary integration strategies have emerged as critical to device advancement: monolithic integration and heterogeneous three-dimensional (3D) integration: (i) Monolithic integration is a mature and cost-effective post-CMOS process that involves the direct fabrication of microbolometer infrared focal plane arrays (IRFPAs) on pre-processed silicon readout integrated circuit (ROIC) wafers. (ii) Heterogeneous 3D integration, by contrast, has garnered increasing attention for its unique ability to combine dissimilar materials, structural elements, and functional components through co-design and advanced micro/nano-fabrication techniques. This approach offers enhanced design flexibility and performance scalability, making it a promising route for next-generation uncooled infrared imaging systems.

**Figure 18:**
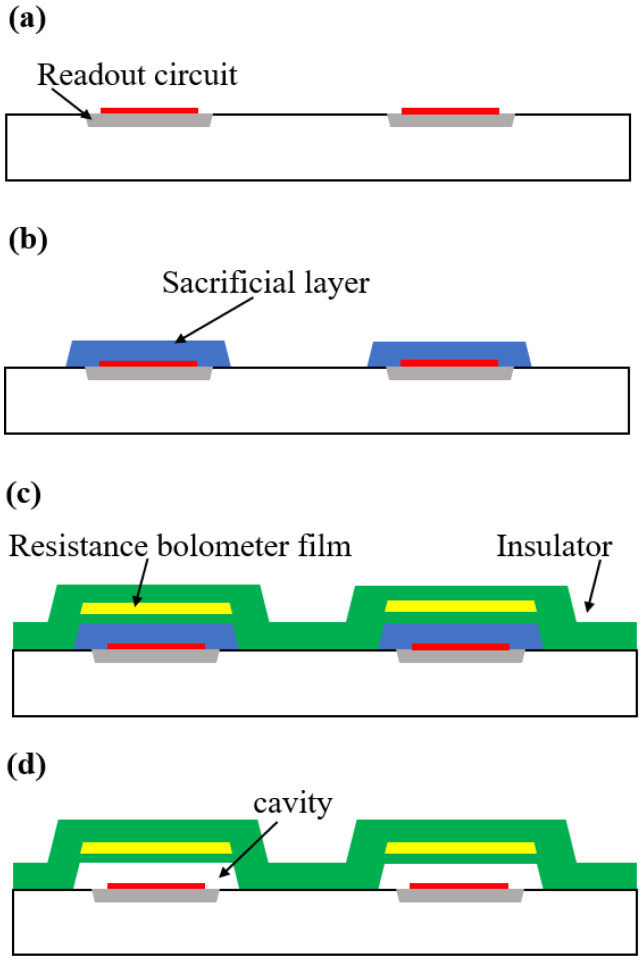
Process flow for monolithic integration of uncooled FPA bolometers: (**a**) readout circuit; (**b**) sacrificial layer formation; (**c**) resistance bolometer film and insulator formation; (**d**) cavity formation.

Monolithic integration, a cost-effective and well-established post-CMOS process, remains the most widely adopted fabrication approach for modern uncooled IRFPA microbolometers. In this method, microbolometer structures are directly fabricated on pre-processed silicon ROIC wafers. The ROIC provides the necessary biasing for the bolometer elements and facilitates the readout of the detected infrared signals. Figure 18 illustrated the fundamental process flow for fabricating resistive uncooled IRFPAs microbolometer via monolithic integration. To begin with, a reflective layer is deposited on a Si wafer imprinted with a Si readout integrated circuit (Figure 18a). The reflective layer commonly utilizes metal materials like Ti or Al and is patterned through photolithography technology. Subsequently, a sacrificial layer is deposited and patterned to form mesas (Figure 18b). There are a variety of materials suitable for sacrificial layers, including organic materials, inorganic materials, semiconductors, and metals. Polyimide is the most frequently used sacrificial layer material in the fabrication of commercial microbolometer FPAs since it can be selectively removed by dry etching processes like the oxygen plasma etching technique. Thereafter, a multilayer membrane structure of the microbolometer is deposited and patterned on the sacrificial mesa to form the microbridge (Figure 18c). The multilayer structure typically encompasses a support layer, a thermistor layer, an electrode film layer, an infrared absorbing layer, and a passivation layer. Finally, the sacrificial mesa is removed by selective etching to release the microbridge (Figure 18d). It is noteworthy that high-temperature processes exceeding 450 °C are generally not permissible for the monolithic integrated fabrication of microbolometer FPAs, as excessive temperatures can damage the Si readout integrated circuit [144]. All current large-scale production commercial VO_x_, TiO_x_, and α-Si resistive IRFPA microbolometers are fabricated through monolithic integration.

**Figure 19:**
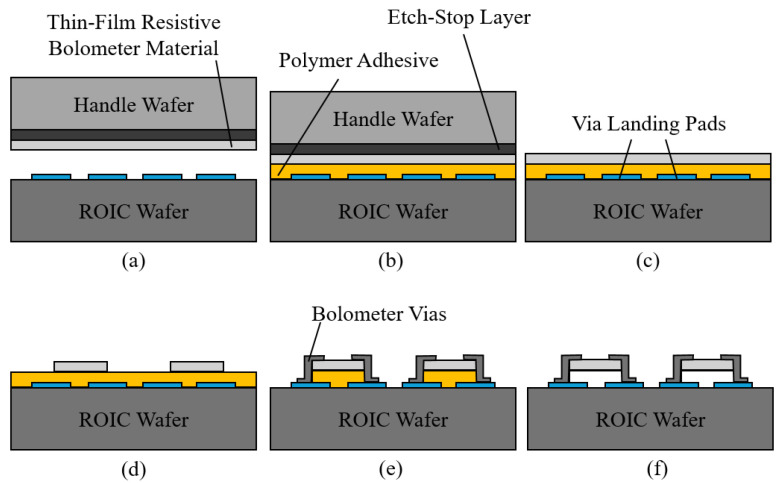
Process flow for heterogeneous 3D integration of uncooled FPA bolometers: (**a**) handle wafer and ROIC wafer; (**b**) bonding for handle wafer and ROIC wafer; (**c**) via landing pads formation; (**d**) pad formation; (**e**) bolometer vias; (**f**) cavity and device formation.

Heterogeneous 3D integration has garnered significant interest from both industry and academia due to its capability to integrate diverse materials, device structures, and functional components through co-design and advanced micro-/nano-fabrication techniques. In recent years, this approach has been increasingly explored for the fabrication of uncooled FPA microbolometers, offering new opportunities for enhanced performance, design flexibility, and functional integration beyond the limitations of conventional monolithic processes [233], and the fundamental process flow is illustrated in Figure 19. First, the thermistor material is deposited on a handle wafer with an etch-stop layer. Then, the handle wafer and Si readout integrated circuit wafer are bonded together using low-temperature polymer bonding (Figure 19b). Here, polymer functions not only as an adhesive but also as a sacrificial layer. After the removal of handle wafer by an etching process, the thermistor material is transferred from the handle wafer to the readout integrated circuit wafer (Figure 19c). The subsequent process steps largely mirror those employed in monolithic integration for fabricating FPA microbolometers. However, a key distinction in 3D heterogeneous integration lies in the separation of the thermistor material and the silicon ROIC onto different wafers. This decoupling allows for the use of high-temperature deposition and annealing processes to optimize the properties of the thermistor material without compromising the integrity of the ROIC. Additionally, materials that are challenging to implement in monolithically integrated FPA microbolometers, such as quantum-well or single-crystalline thermistor materials, can be more readily incorporated, thereby expanding the design space and functional potential of next-generation devices [234].

### 4.3. Development of IRFPA Microbolometers

**Figure 20:**
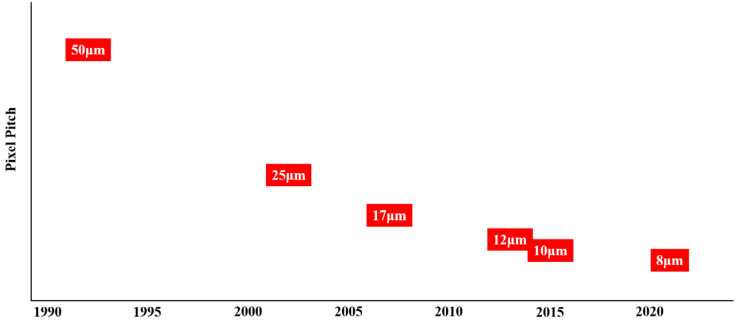
Pixel pitch for microbolometers.

Uncooled thermal imagers have garnered growing interest in recent years due to their broad applicability across various domains. A key developmental focus in this field is the continuous reduction in the pixel pitch to enable higher spatial resolution, as shown in Figure 20. Notably, significant progress has been achieved in FPA technologies, largely driven by advancements in thermistor material systems. This section highlights recent developments in FPAs, categorized by thermistor material type. Among these, hydrogenated α-Si remains one of the most commonly employed thermistor materials in infrared FPA microbolometers. However, the pixel size reduction in α-Si-based microbolometers introduces several limitations that adversely impact the device sensitivity. First, a reduced pixel area decreases the effective absorption surface, thereby limiting the amount of incident infrared power captured. Second, the downsizing of the sensing volume leads to a relative increase in *1/f* noise, which degrades the signal fidelity. Third, the reduction in the thermal isolation region accelerates heat dissipation to the substrate, ultimately diminishing the thermal sensitivity.

**Figure 21:**
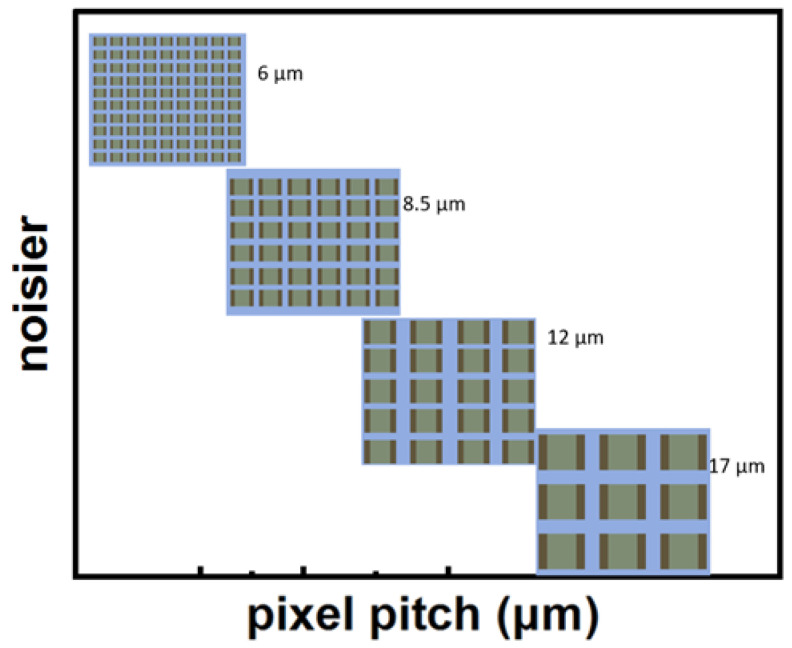
It is observable that as the pixel pitch decreases, the background tends to become noisier.

To address these challenges, Fraunhofer IMS has, since 2016, focused on the development of uncooled α-Si-based FPA microbolometers incorporating vertical nanotube structures for improved thermal isolation. This design strategy aims to preserve the sensitivity while enabling continued pixel miniaturization [235,236,237,238]. This structure comprises an IR-sensitive membrane and two vertical nanotubes, which function as electrical contacts to the ROIC. This particular design enables the independent manipulation of the electrical contact area, irrespective of the pixel pitch. According to this technological concept, Fraunhofer IMS has already presented FPA microbolometers possessing complete electro-optical functionality, featuring pixel sizes varying from 17 µm to as small as 6 µm (Figure 21) [239]. According to their evaluation, considering the further enhancements of sensing layer and nanotube material, proposed nanotube microbolometers display the potential to successfully attain uncooled thermal imagers with pixel pitches reaching the optical detection limit in the LWIR.

**Figure 22:**
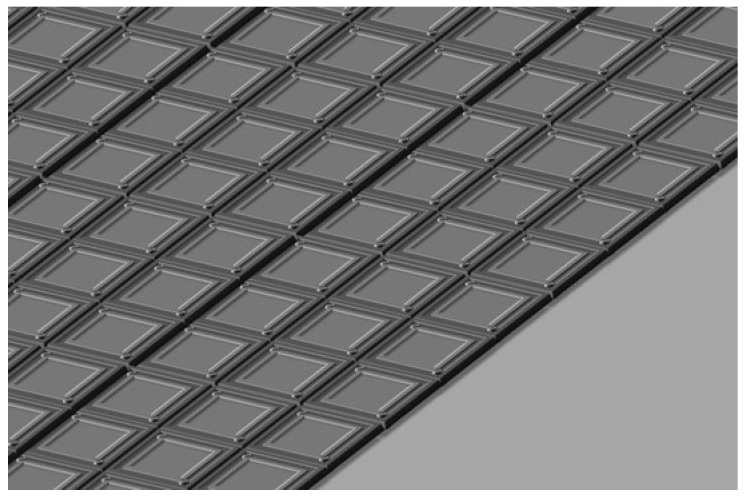
Schematic image of the FPA microbolometer that is seamlessly integrated onto an ROIC.

VO_x_ is another widely adopted thermistor material in FPA microbolometers and has undergone substantial technological refinement in recent years. Intensive research has been dedicated to tailoring its microstructure and stoichiometry to enhance the thermosensitive properties such as the TCR, thermal stability, and noise performance. One notable advancement comes from ASELSAN, which developed a dual-layer pixel architecture designed to achieve a high fill factor, thereby improving both the infrared absorption efficiency and optical responsivity. In this architecture, the lower pixel layer features a suspended arm structure thermally isolated from the ROIC via an air cavity. The upper layer comprises a W-doped VO_x_ multiphase thin film that serves as the infrared-sensitive element, encapsulated by electrodes, an infrared absorber layer, and passivation layers. Furthermore, the thermal sensitivity of the microbolometer is enhanced by minimizing the heat loss from the thermally active regions to the heat sink. The SAFIR640 device, fabricated on 8-inch CMOS ROIC wafers designed by ASELSAN, exemplifies the successful implementation of this advanced pixel structure through scalable microfabrication techniques [240]. A double layer 17 µm pitch microbolometer structure with an active material layer of VO_x_ has been designed (Figure 22). The SAFIR640 detectors, featuring a 640 × 480 array format, exhibit high TCR and low noise characteristics. Experimental evaluations demonstrate that these detectors achieve low NETD values and short thermal time constants. Thermal isolation within the SAFIR640 pixel is realized through suspended microbridge or arm structures positioned beneath the active bolometric layer. The entire microbolometer pixel array is monolithically integrated onto an 8-inch CMOS ROIC wafer. Through precise control of deposition parameters and post-deposition treatments, the TCR of VO_x_ has been further enhanced, yielding improved sensitivity to infrared radiation. Moreover, the integration of VO_x_ with advanced MEMS fabrication techniques has facilitated the realization of FPAs with reduced pixel pitches and increased fill factors, thereby significantly improving the spatial resolution and imaging performance. This integration strategy not only optimizes microbolometer device characteristics but also underpins the advancement of next-generation infrared imaging systems.

**Figure 23:**
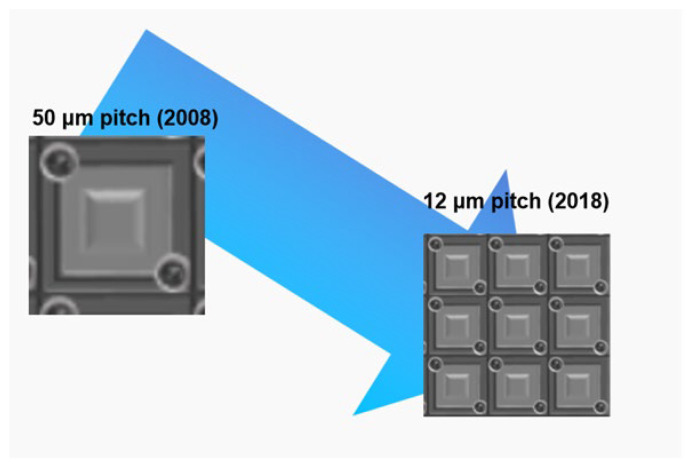
Technical advancement of the TiO_x_-based FPA microbolometer at i3system over the years of 2008 to 2018.

In addition to α-Si and VO_x_, TiO_x_ has emerged as a promising thermistor material owing to its tunable room-temperature resistivity and temperature-dependent resistance characteristics. Doping TiO_x_ with elements such as antimony (Sb), boron (B), and phosphorus (P) has been demonstrated to substantially enhance its negative temperature coefficient (NTC) behavior. Furthermore, the intrinsic properties of TiO_x_, including its relatively high conductivity coefficient and large dielectric constant, contribute to reinforcing the NTC effect, making it highly suitable as a thermistor material. Notably, TiO_x_ is favored for its combination of low *1/f* noise and high TCR. Motivated by these advantageous characteristics, i3system has focused its efforts on uncooled infrared sensor development utilizing TiO_x_-based thermistors. Since 2008, the company has pursued progressive pixel pitch reduction through several developmental phases, advancing the state-of-the-art in TiO_x_-based microbolometer technology [193,198,241]. During the development of the 8 μm pixel pitch microbolometer, numerous challenges were encountered. From a pixel design perspective, to address the limitations inherent in the 8 μm pixel FPA, a 0.18 μm CMOS technology was employed for the ROIC, coupled with a four-channel integration architecture to enhance the overall device performance (Figure 23). Regarding the fabrication process, difficulties associated with photoresist thickness control during the formation of 8 μm structures were effectively overcome. Multiple strategies, including initial thermal isolation pattern etching, were implemented to ensure the structural integrity and successful patterning. Performance evaluation metrics, such as the NETD and thermal time constant, demonstrated that despite a reduction in the figure of merit (FOM) compared to single-layer structures, the 8 μm FPA achieved an average NETD of 45 mK and a thermal time constant of 11 ms, satisfying the targeted specifications. Environmental testing further confirmed the robustness and stability of the device architecture. The key innovations underpinning this research can be summarized as follows: (I) technological integration leveraging a 0.18 μm CMOS ROIC design alongside 0.18 μm MEMS fabrication to realize an umbrella-structured FPA that balances high performance with small pixel pitch, effectively mitigating the thermal isolation and fill factor constraints; (II) material exploitation utilizing TiO_x_ as the thermosensitive layer, capitalizing on its doping-tunable properties, including low *1/f* noise and elevated TCR, distinguishing this approach from conventional infrared sensor materials; and (III) process innovation addressing photoresist thickness challenges via novel process sequencing such as prioritizing thermal isolation pattern etching and incorporating a hard mask, thereby enhancing manufacturability and device performance beyond traditional microbolometer pixel fabrication methods.

**Figure 24:**
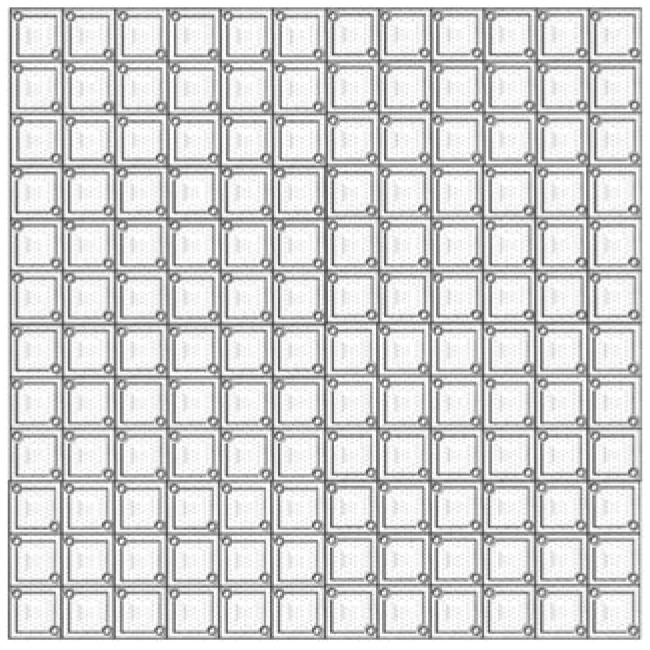
Schematic images of bolometer-type infrared detector: top view of the array elements.

Recently, FPA microbolometers have been increasingly envisioned for deployment in mobile platforms such as drones and automobiles. In pursuit of developing high-sensitivity, cost-effective infrared FPA microbolometers that can serve as alternatives to conventional thermistor materials like α-Si, VO_x_, or TiO_x_, NEC Corporation has proposed single-walled carbon nanotubes (SWCNTs) as a promising candidate. This is attributed to their exceptional physical properties, including high electrical conductivity, superior crystallinity, and robust chemical stability [214,242]. Notably, the TCR is around −6%/K, which is three times that of VO_x_, making them a promising candidate for bolometer materials. The fabrication steps of 640 × 480 pixel bolometer-type uncooled IRFPAs are as follows: (I) a photosensitive area and support legs were formed on the readout integrated circuit (ROIC) substrate using a bottom-up technique; (II) passivation film on the contact pad was removed, and a sacrificial layer, a protective film, and a SiO_2_ film were sequentially formed; (III) an electrode pattern was prepared, and semiconducting SWCNT network film was deposited via a wet process; (IV) an upper protective film was added, and input/output pads were opened; (V) slits were made, and the chip was half-cut; (VI) the sacrificial layer was removed. The resulting semiconducting SWCNT network film was in intimate contact with the TiAlV (TAV) electrode. To evaluate the infrared image sensor, a test elementary group (TEG) was utilized. It was found that responsivity of the sensor increased linearly with the bias voltage, reaching over 10^5^(V/W) at 0.2 V. By applying the formulas for the responsivity and the thermal time constant and considering the measured values and structural design parameters, the TCR value of the SWCNT network film as the thermistor material was estimated to be approximately −5%/K or higher, and the thermal time constant was about 5 ms. This clearly demonstrates that the high TCR of the SWCNT network film has a substantial impact on the responsivity (Figure 24). The detector with high-purity semiconducting SWCNT network films was successfully fabricated through the MEMS process. Looking ahead, the researchers plan to obtain images through ROIC and improve the structure of the light-receiving part to boost the infrared absorption rate, thereby further advancing the development of this technology. With the relentless progression of CMOS technology miniaturization, the performance of the ROIC can be further augmented. The reduction in transistor dimensions and the shortening of interconnect lengths, which are hallmarks of advanced CMOS processes, precipitate a diminution in parasitic capacitances and resistances [243,244,245]. Consequently, the speed and power consumption characteristics of the readout integrated circuit (ROIC) can be precisely optimized. In imaging applications, a more compact and efficient ROIC facilitates the handling of higher data throughput from detectors, thereby improving overall system frame rates and image quality. Furthermore, reduced power consumption is critically important for portable and battery-operated devices, as it extends their operational longevity.

Sustained research and development efforts in thermistor materials have substantially advanced the performance of FPAs for thermal infrared sensing. The distinct physical and electrical properties inherent to each material system, combined with ongoing innovation, have facilitated notable enhancements in sensitivity, resolution, and operational stability across a wide range of thermal imaging applications. Moving forward, future research is expected to emphasize the further optimization of established materials alongside the investigation of novel material systems and hybrid architectures. Such efforts are critical to addressing the growing demand for next-generation thermal infrared sensors that offer ultra-high resolution, improved sensitivity, and robust long-term environmental resilience.

## 5. Conclusions and Future Perspectives

In conclusion, this review has outlined the fundamental principles of microbolometer technology and synthesized recent advances in resistive-type devices, focusing on thermistor materials and FPAs. VO_x_ remains the dominant thermistor material, with enhanced TCR and reduced noise achieved through doping and compositional engineering. Silicon-based alternatives such as phosphorus-doped α-Si:H demonstrate superior TCR performance, while TiO_x_ has gained commercial traction due to its favorable thermal stability and tunable electrical properties. Emerging materials, including GeSn alloys and semiconducting SWCNT networks, exhibit significant potential for next-generation applications by combining high sensitivity with low noise levels. Progress in FPA fabrication has facilitated pixel pitch reduction to 6 μm, enabled by innovations like vertical nanotube thermal isolation and 3D heterogeneous integration of single-crystalline Si with readout circuits. These developments have improved fill factors and responsivity, advancing uncooled microbolometers closer to cooled detector performance [198,246,247]. Nevertheless, scaling down to 5 μm pixels remains challenging, requiring further refinement of thermistor materials, microfabrication techniques, and thermal management strategies [131]. Single-crystalline Si-based semiconductors stand out due to CMOS compatibility and superior noise characteristics, with recent breakthroughs in heterogeneous 3D integration overcoming prior deposition and thermal constraints. Simultaneously, novel materials such as SWCNT networks offer promising avenues for enhancement, though their large-scale commercialization depends on improved mechanical stability and thermal insulation [248,249,250,251,252,253,254,255,256,257,258,259,260]. Collectively, these advancements underscore the critical interplay between material innovation and system-level optimization as key drivers propelling microbolometer technology toward ultra-high resolution, enhanced sensitivity, robust reliability, and wide-ranging applicability in both civil and military infrared imaging domains.

Although this review has primarily focused on thermoresistive materials, it is worth noting the increasing attention toward thermoelectric compounds in uncooled infrared detection. These materials offer the unique advantage of self-powered operation, potentially reducing system-level power consumption. Integrating thermoelectric elements into microbolometer arrays could enable dual-mode sensing capabilities, where both temperature-dependent resistance changes and thermoelectric voltage generation are exploited. Advances in power factor optimization, thermal isolation design, and circuit-level integration are expected to further improve the performance and compatibility of such hybrid systems. While a comprehensive discussion of thermoelectric-based detectors lies beyond the scope of this review, future work exploring synergistic integration of thermoresistive and thermoelectric technologies could open new pathways for high-efficiency multifunctional IRFPAs.

## Figures and Tables

**Table 1:** Reported properties of VO_x_ thermistor material in terms of TCR and resistivity.

Year	Organization	Technique	Materials	TCR (%/K) @RT	Resistivity (Ω·cm)	Refs
2013	NEC	ELAMOD	Vanadium niobate	−3.6	-	[175]
2019	Hacettepe University	DC magnetron sputtering	α-VWO_x_	−2.45 −2.75 −3.02	2.4 4.8 8.1	[176]
2022	Ajou University	ALD	Al-doped VO_x_	−4.2	-	[177]
2023	UNT	DC sputtering	VO_x_/SWCNT composite	−3.65	-	[178]
2024	Ajou University	PEALD	Al_2_O_3_/Al:VO_x_	−3.5	-	[179]

**Table 2:** Reported properties of α-Si thermistor material in terms of TCR and resistivity.

Year	Organization	Technique	Materials	TCR (%/K) @RT	Resistivity (Ω·cm)	Refs
2010	KTH	RPCVD	Si_0_._35_Ge_0_._65_ (C)/Si(C) MQWs	−4.5	-	[74]
2010	L-3	PECVD	α-Si:H	~−3.9	-	[188]
2018	University of Missouri	RF magnetron sputtering	α-Si_0_._054_Ge_0_._877_O_0_._069_	−3.5	629	[189]
2020	INAOE	PECVD	pm-Si_x_Ge_1−x_:H	−4.08	6.67 × 10^4^	[190]
2023	Chungnam National University	PECVD	high SCCM B-doped α-Si:H low SCCM B-doped α-Si:H high SCCM P-doped α-Si:H low SCCM P-doped α-Si:H	−3.01 −4.16 −5.01 −5.08	2.28 × 10^3^ 3.45 × 10^3^ 1.49 × 10^5^ 2.48 × 10^6^	[191]

**Table 3:** Reported properties of TiO_x_ thermistor material in terms of TCR and resistivity.

Year	Organization	Technique	Materials	TCR (%/K) @RT	Resistivity (Ω·cm)	Refs
2008	i3 system Company	reactive sputtering	TiO_2−δ_	−2.1	1	[193]
2015	KAIST	RF reactive magnetron sputtering	TiO_2−x_ @R_O2_ = 3.7%	−3.65	42.65	[194]
2016	ASTU	ALD	TiO_x_	−7.2	4.5 × 10^−4^	[195]
2018	KAIST	RF reactive sputtering	0 at.% Nb:TiO_2−x_ 0.5 at.% Nb:TiO_2−x_ 1 at.% Nb:TiO_2−x_	−2.54 −2.65 −2.78	0.82 0.75 0.69	[196]
2022	Solid State Physics Laboratory	RF reactive magnetron sputtering	TiO_1_._5_ TiO_1_._9_	−1.12 −4.87	1.2 58.7	[197]

**Table 4:** Reported properties of other thermistor material in terms of TCR and resistivity.

Year	Organization	Technique	Materials	TCR (%/K) @RT	Resistivity (Ω·cm)	Refs
2003	INAOE	LP-PECVD	α-Ge	−5	400	[199]
2015	Rensselaer Polytechnic Institute	drying solution	Semiconductive SWNT Semi-Metal SWNT Metallic SWNT	−6.5 ± 1.9 −2.3 ± 0.9 −3.0 ± 0.4	1.1 × 10^6^ 4.8 × 10^5^ 5.3 × 10^5^	[200]
2017	Universidad Autónoma de San Luís Potosí	drying solution	t-MWNT	−5.6 ± 0.1	-	[201]
2017	KAIST	RF magnetron sputtering	Poly-Ni_1−x_O at 1 mTorr Poly-Ni_1−x_O at 2 mTorr Poly-Ni_1−x_O at 5 mTorr Poly-Ni_1−x_O at 10 mTorr	−2.76 −2.82 −3.80 −5.62	10.22 13.35 205.34 4.55 × 10^4^	[202]
2018	KAIST	RF magnetron sputtering	(Li_y_Ni_1−y_)_1−x_O	−2.78	-	[203]
2023	King Saud University	co-sputtering	Ge Ge_0_._83_Sn_0_._17_ Ge_0_._78_Sn_0_._22_ Ge_0_._75_Sn_0_._25_ Ge_0_._69_Sn_0_._31_ Ge_0_._65_Sn_0_._35_ Ge_0_._6_Sn_0_._4_	−4.45 −3.96 −3.63 −3.29 −3.12 −2.74 −2.52	487.2 164.6 69.14 45.46 27.98 9.23 4.86	[204]

## Data Availability

No new data were created or analyzed in this study.
